# Reliability and construct validity of the Hungarian version of Skindex-Mini

**DOI:** 10.1371/journal.pone.0350749

**Published:** 2026-06-23

**Authors:** Borbála Német, Balázs András Varga, Norbert Kiss, Uğur Çakır, Miklós Sárdy, Adrien Rigó

**Affiliations:** 1 Doctoral School of Psychology, ELTE Eötvös Loránd University, Budapest, Hungary; 2 Institute of Psychology, ELTE Eötvös Loránd University, Budapest, Hungary; 3 Department of Dermatology, Venereology and Dermatooncology, Semmelweis University, Budapest, Hungary; Bogomolets National Medical University: Nacional’nij medicnij universitet imeni O O Bohomol’ca, UKRAINE

## Abstract

The Skindex-Mini is a brief 3-item dermatology-specific quality-of-life tool, that has been previously validated in English and Spanish. This study evaluated the reliability and construct validity of the Hungarian version in patients with skin diseases. In a cross-sectional study, 578 patients with inflammatory or noninflammatory conditions completed the Hungarian Skindex-Mini alongside established measures including the Dermatology Life Quality Index–Revised, Difficulties in Emotion Regulation Scale-16, and Stigmatization Scale for Chronic Illnesses–8 Questionnaire. Validity was assessed using and Spearman correlations. Confirmatory factor analysis supported a unidimensional structure, while Item Response Theory analyses indicated strong item discriminatory power and measurement invariance across groups. The tool demonstrated moderate convergent validity with stigma and distress, and acceptable discriminant validity with well-being. The Hungarian Skindex-Mini exhibits robust psychometric properties, supporting its use for rapid screening of dermatology-related quality-of-life burden. Its brevity makes it suitable for diverse clinical and research applications.

## Introduction

Chronic dermatological conditions impose a multidimensional burden that extends beyond physical symptoms to encompass profound psychosocial consequences [1]. The visible nature of skin diseases frequently precipitates stigmatization, emotional distress, and functional impairment, with patients exhibiting elevated rates of depression (30–40%), anxiety (20–30%), and social withdrawal compared to healthy controls [2]. This complex disease burden necessitates robust patient-reported outcome measures (PROMs) to capture health-related quality of life (HRQoL) in both clinical and research settings [3–5].

While comprehensive instruments, including the Skindex-29 Quality of Life Questionnaire (Skindex-29 QoL) [6] and Dermatology Life Quality Index–Revised (DLQI-R; DLQI-R) [7,8], provide detailed HRQoL assessments, their length (29 and 10 items, respectively) limits their use in routine practice [9]. The Skindex-Mini brief Quality of Life (QoL) Measure for patients with skin diseases [9] (Skindex-Mini) addresses this challenge as a 3-item ultrashort form that retains the psychometric rigor of its parent instruments while optimizing clinical utility [10]. Each item evaluates a core HRQoL domain: the first captures symptom burden (itching, pain), the second evaluates emotional impact (worry, embarrassment), and the third assesses functional limitations. Table 1 summarizes existing validation studies, including sample sizes, conditions treated, and psychometric findings. This includes the language of the instrument, sample size, the dermatologic conditions studied, statistical methods applied, and a brief summary of psychometric findings for the Skindex-Mini in that context [9–13]. Each study suggests that the 3-item Skindex-Mini is a reliable and valid ultra-brief tool for assessing dermatology-related quality-of-life burden, with strong correlations to established longer instruments and sensitivity to differences in disease severity or treatment effects.

**Table 1:** Summary of peer-reviewed studies evaluating the Skindex-Mini’s validation and psychometric properties across languages and populations.

Study (Citation)	Language	Sample Size	Skin Disease Context	Statistical Methods	Validity	Reliability	Conclusion
Swerlick et al., 2021 – Initial Skindex-Mini development and validation (USA).	English (also translated to Spanish)	Not reported (Pilot sample of adult dermatology patients)	Mixed dermatologic conditions (adult patients in general dermatology clinics).	– Item selection based on psychometric criteria (1 item per domain from Skindex-16).– Correlation analysis comparing Skindex-Mini scores with original Skindex-16.	High correlation between 3-item Skindex-Mini and Skindex-16 overall scores (reported qualitatively as preserving Skindex-16 validity). Skindex-Mini domain items were chosen for maximal representativeness of each QoL domain, ensuring strong content validity.	The three items together showed acceptable internal consistency (not numerically reported, but implied).	Authors note the Skindex-Mini retained the reliability and validity of the longer instrument while improving feasibility in clinic.
Orenstein et al., 2020 – Validation in Hidradenitis Suppurativa patients (Emory Univ.).	English	75 HS patients (108 clinical encounters)	Hidradenitis Suppurativa (chronic inflammatory skin disease).	Pearson correlation coefficients between Skindex-Mini and Skindex-16 domain scores.– Secondary correlations with itch (NRS) and pain scores.	Concurrent validity was excellent – Skindex-Mini domain scores correlated r = 0.77–0.80 with corresponding Skindex-16 domains (Symptoms, Emotions, Function, all p < 0.001). The Skindex-Mini captured QoL impairment similar to the full Skindex-16 in HS patients. It also correlated positively with itch and pain ratings, aligning with symptom burden.	With only one item per domain, traditional Cronbach’s α was not applicable by domain; however, the strong domain correlations serve as evidence of measurement reliability/accuracy.	The 3-item Skindex is a “streamlined QoL instrument” that can be implemented in routine care for HS, even in a racially diverse sample.
Yeung et al., 2022 – Use in melanoma patients with immunotherapy side-effects (Emory/Duke VA).	English	18 melanoma patients (receiving PD-1/PD-L1 immunotherapy)	Cutaneous adverse effects from immune checkpoint inhibitor therapy in advanced melanoma.	– Descriptive statistics of Skindex-Mini domain scores.– Group comparisons (patients with significant cutaneous toxicity vs. expectations/norms).	Known-groups validity demonstrated – patients who developed immune-related skin toxicities had markedly elevated Skindex-Mini Symptom and Function domain scores, indicating worse QoL in those domains. (For example, pruritus, pain and functional limitations due to rash were reflected in higher Skindex-Mini scores.) Emotional domain scores were elevated to a lesser extent, suggesting physical aspects were predominant.	Given the small N, no reliability coefficients reported; instrument was still able to consistently reflect QoL issues in this cohort.	Even in a small sample, Skindex-Mini proved sensitive to QoL impact of drug-induced skin side effects, supporting its utility in oncology dermatology settings.
Dizon et al., 2022 – Validation in Adult Atopic Dermatitis patients (OHSU).	English	156 AD patients; 156 controls (healthy)	Atopic Dermatitis (adults, moderate-to-severe; comparison to healthy controls).	– Internal consistency (Cronbach’s alpha) for 3-item scale.– Convergent validity via correlations with other measures (e.g., itch severity, possibly DLQI or POEM).– Discriminant validity: comparison of patient vs. control scores.	Discriminant validity was strong – mean Skindex-Mini scores were significantly higher in AD patients than in controls (indicating greater QoL impairment in patients, p < 0.001). The Skindex-Mini could clearly distinguish those with eczema from those without. Convergent validity: Skindex-Mini scores correlated with clinical severity (e.g., worse eczema extent/severity and itch intensity corresponded to higher QoL impairment scores, as expected). This aligns with other AD-specific QoL tools, confirming the Skindex-Mini’s relevance.	Cronbach’s α for the total Skindex-Mini was reported and found to be acceptable (despite only 3 items, suggesting the items collectively capture an underlying QoL impact).	The Skindex-Mini showed robust psychometric properties in adults with atopic dermatitis, supporting its use for quick QoL assessment in eczema. Its brevity and validity make it attractive for both clinical practice and research in dermatology.
Sheth et al., 2023 – Validation in Pediatric Atopic Dermatitis patients (Northwestern Univ.).	English	N not explicitly stated (pediatric AD patients in a dermatology clinic; presumed ~50–100 based on typical study)	Atopic Dermatitis in children (ages not given, but likely school-age)	– Known-groups comparison by disease severity (mild/moderate/severe AD as per vIGA and EASI scores).– Concurrent validity check with other pediatric QoL measures (implied).	The Skindex-Mini Total Score differentiated between mild, moderate, and severe pediatric AD groups (p < 0.05 for trend), demonstrating excellent known-groups validity. Children with more severe eczema had higher Skindex-Mini scores (greater QoL impact). Additionally, Skindex-Mini correlated strongly with other patient-reported outcomes in children with AD (e.g., presumably with the Children’s DLQI or symptom scores), indicating good concurrent validity in the pediatric context.	The study did not emphasize Cronbach’s alpha (given the multi-domain nature and only 3 items), but the instrument was reliable in capturing QoL since even young patients could understand and answer the three questions consistently.	The Skindex-Mini is a valid and child-friendly QoL instrument for pediatric eczema. Its simplicity (only 3 questions) is advantageous for children, and it still yields meaningful distinctions aligned with clinical severity. Authors suggest it could streamline QoL monitoring in pediatric dermatology.

**Abbreviations:** HS = hidradenitis suppurativa; AD = atopic dermatitis; QoL = quality of life; NRS = numeric rating scale; DLQI = Dermatology Life Quality Index; POEM = Patient-Oriented Eczema Measure; vIGA = validated Investigator Global Assessment; EASI = Eczema Area and Severity Index.

Cross-cultural implementation remains underexplored, particularly in linguistically distinct contexts such as Hungarian, where differences in syntax and semantics may affect item interpretation [14]. Beyond methodological considerations, the validation of Skindex-Mini carries substantial clinical implications. Robust and brief PROMs are increasingly required in routine dermatological care to guide treatment decisions, monitor therapeutic outcomes, and support reimbursement processes, particularly in chronic inflammatory conditions such as psoriasis, atopic dermatitis, and hidradenitis suppurativa [3,4].

By establishing the reliability and validity of the Hungarian Skindex-Mini, this study enables the integration of dermatology-specific quality-of-life data into everyday practice, where rapid yet accurate assessments are essential for triage, follow-up, and shared decision-making.

### Study aims and hypotheses

This study aimed to evaluate the psychometric properties of the Skindex-Mini QoL Health Questionnaire in a large sample of adults with dermatological conditions. Specifically, we pursued six complementary objectives.

1. Dimensionality and Consistency: We utilised confirmatory factor analysis (CFA) to test for a unidimensional structure and hypothesised satisfactory internal consistency (ω, α ≥ 0.70).
2. Item Characteristics: We applied Item Response Theory (IRT) to assess item discrimination and coverage of the latent quality-of-life construct.
3. Construct Validity: We hypothesised moderate-to-strong convergent validity with related constructs (e.g., DERS-16, SSCI-8, Distress Thermometer) and acceptable discriminant validity with unrelated measures (e.g., Anger-Out, Body Absorption Scale).
4. Unique Predictors: We used hierarchical regression to identify psychometric predictors of Skindex-Mini scores while controlling for demographic factors.
5. Known-Groups Validity: We tested the instrument’s ability to discriminate between DLQI-R severity bands, expecting scores to increase monotonically with disease impact.
6. Diagnostic Accuracy: We employed receiver operating characteristic (ROC) analyses to determine clinically significant cutpoints and evaluate the tool’s sensitivity, specificity, and discriminatory validity across various dermatological diagnoses.

### Patients and methods

This prospective observational study validated the Hungarian Skindex-Mini. The study adhered to the Declaration of Helsinki and was approved by the Ethics Committees of Eötvös Loránd and Semmelweis Universities (SE RKEB 2182021); all participants provided written informed consent.

Adult native Hungarian speakers with inflammatory, symptomatic, or asymptomatic dermatological diseases were recruited online, and at Semmelweis University, from the 15th of november, 2021, til 20th of July, 2025. Patients unable to comprehend or complete the survey due to literacy or cognitive issues were excluded.

Based on CFA and regression analysis requirements (alpha = 0.05, power = 0.95, medium effect size), a minimum sample size of 200 was established. The regression model included the Difficulties in Emotion Regulation Scale-16, 9-item Beck Depression Inventory, Anger-In/Out scales, Stigmatization Scale for Chronic Illnesses–8 Questionnaire, BAS, DH, WHO-5 Well-Being Index, and Visual Analog Scale (EQ-VAS) of the EuroQol-5D Scale.

### Instruments

To assess the construct validity of the Hungarian Skindex-Mini, participants completed a series of established, validated questionnaires covering health-related quality of life, emotion regulation, stigma, and psychological well-being. For each instrument described below, the internal consistency (Cronbach’s α) was calculated for the current sample and is reported to provide context for the subsequent analyses.

#### Dermatology-specific quality of life.

- **Skindex-Mini QoL Health Questionnaire (Skindex-Mini)** [9] is a 3-item ultrashort measure designed to assess dermatology-specific health-related quality of life (HRQoL). Each item represents a core domain: symptom burden (e.g., “In the past week, how often were you bothered by your skin symptoms...?”), emotional impact (e.g., “...how often did your skin symptoms cause emotional difficulties...?”), and functional limitations (e.g., “...how often did your skin symptoms make it difficult for you to engage in activities...?”). Respondents rate the frequency of each experience over the past week on a 7-point Likert scale (0 = Never to 6 = All the time). Item scores are summed to produce a total raw score (range 3--15), which is then transformed to a 0--100 linear scale, with higher scores indicating greater impairment (worse QoL). The internal consistency in the current sample was good (Cronbach’s α = 0.78).
- **Dermatology Life Quality Index-Revised (DLQI-R)** [7,8] is a 10-item questionnaire that assesses the impact of skin diseases on QoL over the past week. It covers domains such as symptoms and feelings, daily activities, leisure, work or school, personal relationships, and treatment. Items are rated on a 4-point Likert scale (0 = Not at all/Not relevant to 3 = Very much). A sample item is: “Over the last week, how itchy, sore, painful or stinging has your skin been?” The total score ranges from 0 to 30, with higher scores indicating greater QoL impairment. The DLQI-R was used to assess known-groups validity and as an anchor for determining clinically significant cut-points on the Skindex-Mini. In this sample, the DLQI-R demonstrated excellent internal consistency (α = 0.90).

#### Emotion regulation and distress.

- **Difficulties in Emotion Regulation Scale-16 (DERS-16)** [14,15] is a 16-item self-report measure that assesses clinically relevant difficulties in emotion regulation. It comprises five subscales: Non-acceptance of emotional responses, Difficulty engaging in goal-directed behavior, Impulse control difficulties, Limited access to emotion regulation strategies, and Lack of emotional clarity. Items (e.g., “I have difficulty making sense out of my feelings”) are rated on a 5-point Likert scale (1 = Almost never to 5 = Almost always). Total and subscale scores are calculated as the mean of the respective items, with higher scores indicating greater difficulties in emotion regulation. Internal consistency in our sample was excellent for the total score (α = 0.95) and good-to-excellent for the subscales (α range = 0.85–0.94).
- **9-item Beck Depression Inventory-Fast Screen (BDI-FS)** [16,17] is a 9-item screening tool for depressive symptoms in medical patients, excluding somatic items that might be confounded with physical illness. Items (e.g., “Sadness”) are rated on a 4-point scale (0–3), with total scores ranging from 0 to 27. Higher scores indicate more severe depressive symptoms. The internal consistency in this sample was acceptable (α = 0.74).
- **Distress Thermometer (DT)** [18,19] is a single-item, 11-point visual analog scale (0 = No distress to 10 = Extreme distress) that asks patients to rate their average level of psychological distress over the past week, including the current day. Higher scores indicate greater distress. It is widely used in psycho-oncology and psychodermatology as a rapid screening tool. In this study, it was used to assess convergent validity (α is not applicable for a single-item measure).

#### Stigma and social functioning.

- **Stigmatization Scale for Chronic Illnesses--8 Questionnaire (SSCI-8)** [20] is an 8-item measure that assesses perceived or internalized stigma related to having a chronic illness. Items (e.g., “Because of my illness, I felt left out of things”) are rated on a 5-point Likert scale (1 = Never to 5 = Always). A total score is calculated as the mean of all items, with higher scores indicating a greater degree of perceived stigmatization. The scale showed good internal consistency in the current sample (α = 0.89).
- **Body Absorption Scale (BAS)** [21] is a 10-item instrument that measures an individual’s tendency to focus deeply on internal bodily sensations. Items (e.g., “If there is something wrong with my body, I can feel it immediately”) are rated on a 5-point Likert scale (1 = Not true to 5 = Very true). A total score is calculated as the mean of all items, with higher scores indicating greater body absorption. It was included to assess discriminant validity, as it is conceptually distinct from HRQoL. In this sample, the BAS demonstrated acceptable internal consistency (α = 0.73).

#### Psychological well-being and anger expression.

- **WHO-5 Well-Being Index (WBI-5)** [22,23] is a 5-item scale that measures positive psychological well-being. Items (e.g., “I have felt cheerful and in good spirits”) refer to the previous two weeks and are rated on a 6-point Likert scale (0 = At no time to 5 = All of the time). The raw total score (range 0--25) is multiplied by 4 to yield a percentage score from 0 to 100, with higher scores indicating better well-being. It was used to assess divergent validity. The internal consistency was good in this sample (α = 0.83).
- **Anger Expression Scale (AX Scale)** [24] is a widely used measure of anger expression. For this study, we used two subscales: Anger-In (suppression of angry feelings) and Anger-Out (expression of anger towards others or objects). Items are rated on a 4-point Likert scale (1 = Almost never to 4 = Almost always). Subscale scores are calculated as the mean of their respective items, with higher scores indicating a greater tendency to suppress (Anger-In) or express (Anger-Out) anger. These subscales were used to assess convergent (Anger-In) and discriminant (Anger-Out) validity. In our sample, both subscales showed acceptable internal consistency (Anger-In: α = 0.76; Anger-Out: α = 0.70).

#### General health status.

- **Visual Analog Scale (EQ-VAS) of the EuroQol-5D Scale (EQ-5D)** [8,25] is a standard vertical 20 cm visual analogue scale for recording an individual’s self-rated health on a scale from 0 (“The worst health you can imagine”) to 100 (“The best health you can imagine”). It provides a simple, global measure of current health status. Higher scores indicate better perceived health.

### Statistical analysis

All analyses were performed using MPlus 7.1 [26], Stata 14.0 [27], and SPSS 29 [28] for data cleaning and filtering procedures. For the IRT analysis and the ROC curve, we excluded cases with missing values. For regression analyses, we used listwise deletion, and for correlation analyses, we used pairwise deletion. Descriptive statistics were first computed for all variables. Continuous variables (e.g., age) were assessed for normality using the Shapiro–Wilk test, while categorical variables were summarized by frequency and percentage. As several variables violated assumptions of normality, non-parametric methods and robust estimators were applied where appropriate.

#### Dimensionality and internal consistency (Hypothesis 1).

The unidimensional factor structure of the Skindex-Mini was tested using confirmatory factor analysis (CFA) within the framework of structural equation modelling (SEM) in MPlus. A diagonally weighted least squares (DWLS) estimator was applied, as it is well suited for ordinal data. Standardized factor loadings ≥ 0.60 were considered acceptable, while internal consistency was evaluated with Cronbach’s α and McDonald’s ω, with values ≥ 0.70 interpreted as satisfactory.

#### Item-level psychometrics (Hypothesis 2).

To evaluate measurement precision at the item level, we conducted an Item Response Theory (IRT) analysis using the Graded Response Model (GRM). Discrimination and threshold parameters were estimated for each item. Although IRT typically requires a minimum of five items to ensure stable estimates, reporting outcomes for the three-item scale was considered informative. Test information curves (TIF) and test characteristic curves (TCC) were also inspected to determine the range of the latent trait most reliably assessed.

#### Construct validity (Hypothesis 3).

Construct validity was examined via correlation and covariance analyses. Convergent validity was tested by calculating Spearman’s rank-order correlation coefficients between Skindex-Mini scores and theoretically related constructs, including DLQI-R, DERS-16 and its subscales, SSCI-8, DH Types Total, and BDI-FS Total. Discriminant validity was assessed against conceptually distinct constructs (Anger-Out subscale of the AX Scale; BAS Total). Correlation magnitudes were interpreted using thresholds commonly applied in clinical quality-of-life research in dermatology (BJD, Garg et al., 2025; Kirby et al., 2025): coefficients ≥ 0.40 indicated adequate convergent validity, while coefficients ≤ 0.25 indicated adequate discriminant validity. Correlation strength was further described as low or weak correlation (0.10), moderate (0.3), and high (0.5-).

#### Unique predictors of Skindex-Mini scores (Hypothesis 4).

Hierarchical multiple regression analyses were performed to examine the unique contributions of sociodemographic and psychometric predictors to Skindex-Mini scores. Sex and age were entered in the first block, followed by psychometric variables (DERS-16, SSCI-8, AX subscales, BAS, DH Types Total, BDI-FS, WBI-5, EQ-VAS) in the second block. Variance Inflation Factor (VIF) values were checked to assess multicollinearity, and model assumptions were verified using Cook’s distance, Durbin–Watson statistics, and residual diagnostics.

#### Known-groups validity (Hypothesis 5).

To evaluate whether the Skindex-Mini discriminated between DLQI-R severity categories (no effect, small, moderate, very large, extremely large effect), non-parametric Kruskal–Wallis tests were conducted. Post hoc pairwise comparisons were performed with Bonferroni correction. Effect sizes were estimated using epsilon squared (ε²), interpreted as small (≥ 0.01), moderate (≥ 0.06), or large (≥ 0.14).

#### Diagnostic accuracy (Hypotheses 6–7).

Receiver Operating Characteristic (ROC) curve analyses were conducted to assess the ability of the Skindex-Mini total score and its individual items to discriminate between cases with clinically significant dermatology-specific quality-of-life impairment, defined by DLQI-R severity bands (very large and extremely large effect combined). The Area Under the Curve (AUC) was used as the global indicator of accuracy, with values ≥ 0.80 reflecting good discriminative performance. Sensitivity, specificity, positive predictive value (PPV), and negative predictive value (NPV) were calculated at multiple thresholds. The Youden index (J = sensitivity + specificity – 1) was applied to determine the optimal cutpoint, with J ≥ 0.50 considered acceptable.

## Results

### Description of the sample

The study included two samples. In Sample 1, valid age data were available for 147 participants (5 missing), with a mean age of 45.63 years (SD = 18.99, range = 18–88). In Sample 2, valid age data were available for 392 participants (16 missing), with a mean age of 38.56 years (SD = 13.82, range = 18–87). Across both samples, valid age data were available for 539 participants, with a mean age of 40.49 years (SD = 15.32, range = 18–88), as shown in Table 2.

**Table 2:** Descriptive statistics of age distributions in the samples.

Sample		N	Missing	Mean	Median	SD	Minimum	Maximum
1	Age	147	5	45.63	44	18.995	18	88
2		392	16	38.56	36	13.817	18	87

Valid gender data were available for 560 participants. In the combined sample, 117 participants were male (20.9%), 438 were female (78.2%), and 5 (0.9%) indicated another gender category. Because responses were not mandatory, the number of valid observations varied across variables.

### Psychometric properties of the Skindex-Mini

#### Factor structure and internal consistency of the Skindex-Mini (confirmatory factor analysis).

The unidimensionality and internal consistency of the Skindex-Mini were examined using classical test theory. We did not report model fit indices because the unidimensional three-item Confirmatory Factor Analysis **(**CFA) is a just-identified (saturated) model with zero degrees of freedom, meaning that fit indices cannot be meaningfully interpreted. All factor loadings exceeded the recommended threshold of 0.60, ranging from 0.652 to 0.823.

Descriptive statistics and Confirmatory Factor Analysis (CFA) results for Skindex-Mini items are presented in Table 3 Internal consistency was good, with McDonald’s omega = 0.805 and Cronbach’s alpha = 0.784. Item distributions deviated from normality, consistent with the ordinal nature of Likert-type responses, and this was accounted for in subsequent non-parametric analyses.

**Table 3:** Descriptive statistics and Confirmatory Factor Analysis (CFA) results for Skindex-Mini items.

Item	N	Missing	Mean	CI95%		Median	SD	Min	Max	Shapiro-Wilk W	p-value (SW)	Factor loading (95% CI)	Skewness (SE)	Kurtosis (SE)
**Symptoms (SM1):** In the past week, how often were you bothered by your skin symptoms (e.g., itching, stinging, burning, pain, or skin irritation)?	578	313	4.19	4.03	4.36	5	1.98	0	6	0.83	<0.001	0.652 (0.671–0.774)	−0.79 (0.102)	−0.658 (0.203)
**Emotion (SM2):** In the past week, how often did your skin symptoms cause emotional difficulties such as worry, embarrassment, anxiety, or frustration?	578	313	3.99	3.83	4.16	5	2.05	0	6	0.85	<0.001	0.823 (0.828–0.916)	−0.684 (0.102)	−0.853 (0.203)
**Activity (SM3):** In the past week, how often did your skin symptoms make it difficult for you to engage in activities such as going out, pursuing your goals, working, or interacting with others?	578	313	3.23	3.04	3.41	4	2.25	0	6	0.87	<0.001	0.752 (0.752–0.842)	−0.192 (0.102)	−1.436 (0.203)

#### Item Response Theory (IRT) analysis.

To complement the CFA and further evaluate item-level performance, we applied an Item Response Theory approach using the Graded Response Model (GRM). The aim was to assess the discrimination and threshold parameters of each item, thereby determining how effectively the Skindex-Mini captures the latent construct across the full continuum of quality-of-life impairment. Discrimination parameters were 1.89 (Symptoms), 3.53 (Emotions), and 2.31 (Activity), all above the conventional threshold of 1.0, indicating adequate to high ability to differentiate respondents along the latent trait. Threshold estimates revealed that items provided the most information at lower to average levels of the construct (θ approximately −2 to +1), with the second (Emotion) item contributing the most information overall. Item information curves confirmed that the scale is most precise in assessing mild to moderate impairment, whereas measurement precision diminishes for higher severity levels. These results indicate that the Skindex-Mini, in its current form, is well suited for identifying lower-to-mid-range impairments, although additional items may be required to improve coverage at the severe end of the spectrum.

To further examine the contribution of individual items to the overall scale, we also calculated Spearman correlations and corrected item–total correlations. Spearman correlations indicated that all three items were strongly associated with the total Skindex-Mini score. Corrected item–total correlations ranged from 0.79 to 0.87 (Symptoms: ρ = 0.788; Emotion: ρ = 0.840; Activity: ρ = 0.870; all p < .001, df = 576), indicating that each item made a substantial contribution to the total score. Inter-item correlations were moderate to strong (ρ = 0.505–0.612; p < .001) but lower than the item–total correlations, suggesting that although the items are related, they are not redundant and capture somewhat different aspects of the construct.

#### Descriptive statistics of study variables.

Descriptive statistics for the Skindex-Mini and related constructs are presented in Table 4 Skindex-Mini scores in this sample ranged from 3 to 21, with a mean of 14.4 (SD = 5.26), indicating, on average, a moderate level of dermatology-specific quality-of-life impairment. Among the convergent validity measures, the DERS-16 subscales showed mean values between 2.21 and 2.83 on a 1–5 scale, suggesting mild-to-moderate difficulties in emotion regulation. Perceived stigma, from SSCI-8 scores were generally low to moderate (M = 2.06, SD = 0.88). Divergent and discriminant validity measures also showed wide variability: anger expression scores (HD Anger In and Out) ranged from 1.13 to 4.00, BAS from 1.21 to 5.00, and well-being (WBI-5) from 1.0 to 4.0. Psychological distress and depression measures (DH Total and BDI-FS) exhibited higher variability, reflecting heterogeneity in psychological symptom burden across participants. These descriptive patterns provide the context for subsequent correlation analyses examining the associations between Skindex-Mini scores and related constructs. These descriptive findings provide the basis for examining the bivariate associations between the Skindex-Mini and related constructs, which are presented in the subsequent correlation analyses. Descriptive statistics for Skindex-Mini and related constructs is presented below, in Table 4. Spearman correlation matrix for Skindex-Mini and related constructs are presented in Table 5.

**Table 4:** Descriptive statistics for Skindex-Mini and related constructs.

Variable	N	Mean	SD	Median	Range
**Skindex-Mini**	578	14.4	5.26	15.0	3–21
**Skindex-Mini (%)**	578	63.4%	29.2%	66.7%	1-100%
**DERS-16 Total**	551	2.51	0.89	2.44	1–5
**DERS-16 Non-Accept.**	551	2.50	1.17	2.33	1–5
**DERS-16 Goal Dir. Behav.**	551	2.83	1.08	2.67	1–5
**DERS-16 Imp. Contr. Diff.**	551	2.31	1.11	2.00	1–5
**DERS-16 Em. Regul.**	551	2.56	1.07	2.40	1–5
**DERS-16 Lack Em. Clarity**	551	2.21	0.97	2.00	1–5
**SSCI-8**	549	2.06	0.88	1.88	1–5
**HD Anger In**	515	2.72	0.56	2.75	1.13–4
**HD Anger Out**	515	1.99	0.60	2.00	1–4
**DH Types Total**	137	11.30	6.83	10.0	0–39
**BD Total**	141	14.4	4.54	13.0	9–26
**WBI-5 Total**	569	2.51	0.68	2.60	1–4
**BAS Total**	546	3.38	0.72	3.47	1.21–5

**Note:** Variable abbreviations: Skindex-Mini = Skindex-Mini QoL Health Questionnaire; DERS-16 Total = Difficulties in Emotion Regulation Scale-16 total score; DERS-16 Non-Accept. = Non-acceptance of emotional responses; DERS-16 Goal. Dir. Behav. = Difficulties engaging in goal-directed behaviour; DERS-16 Imp. Contr. Diff. = Impulse control difficulties; DERS-16 Em. Regul. = Limited access to emotion regulation strategies; DERS-16 Lack. Em. Clarity = Lack of emotional clarity; SSCI-8 = Stigmatization Scale for Chronic Illnesses–8; HD Anger In = Anger-In subscale of the Anger Expression Scale; HD Anger Out = Anger-Out subscale of the Anger Expression Scale; DH Types Total = Distress Thermometer total score; BD Total = Beck Depression Inventory-Fast Screen total score; WBI-5 Total = WHO-5 Well-Being Index total score; BAS Total = Bodily Absorption Scale total score.

**Table 5:** Spearman correlation matrix for Skindex-Mini and related constructs.

Variable	Skindex-Mini	DERS-16 Total	DERS-16 Non-Accept.	DERS-16 Goal Dir. Behav.	DERS-16 Imp. Contr. Diff.	DERS-16 Em. Regul.	DERS-16 Lack Em. Clarity	SSCI-8	HD Anger In	HD Anger Out	DH Types Total	BD Total	WBI-5 Total	BAS Total
**Skindex-Mini**	1.0	0.23	0.19	0.16	0.15	0.21	0.25	0.43	−0.09	−0.0	0.29	0.24	−0.25	0.21
**DERS-16 Total**	0.23	1.0	0.84	0.82	0.77	0.93	0.5	0.4	−0.35	0.31	0.35	0.47	−0.27	0.16
**DERS-16 Non-Accept.**	0.19	0.84	1.0	0.57	0.52	0.78	0.35	0.36	−0.34	0.17	0.23	0.39	−0.2	0.14
**DERS-16 Goal Dir. Behav.**	0.16	0.82	0.57	1.0	0.61	0.7	0.32	0.29	−0.28	0.23	0.22	0.3	−0.16	0.17
**DERS-16 Imp. Contr. Diff.**	0.15	0.77	0.52	0.61	1.0	0.62	0.29	0.33	−0.14	0.55	0.23	0.39	−0.2	0.07
**DERS-16 Em. Regul.**	0.21	0.93	0.78	0.7	0.62	1.0	0.41	0.37	−0.39	0.25	0.36	0.47	−0.28	0.16
**DERS-16 Lack Em. Clarity**	0.25	0.5	0.35	0.32	0.29	0.41	1.0	0.25	−0.21	0.05	0.27	0.28	−0.19	0.09
**SSCI-8**	0.43	0.4	0.36	0.29	0.33	0.37	0.25	1.0	−0.23	0.19	0.27	0.47	−0.17	0.13
**HD Anger In**	−0.09	−0.35	−0.34	−0.28	−0.14	−0.39	−0.21	−0.23	1.0	−0.01	−0.08	−0.41	0.1	−0.04
**HD Anger Out**	−0.0	0.31	0.17	0.23	0.55	0.25	0.05	0.19	−0.01	1.0	0.12	0.24	−0.04	0.07
**DH Types Total**	0.29	0.35	0.23	0.22	0.23	0.36	0.27	0.27	−0.08	0.12	1.0	0.4	−0.42	0.31
**BD Total**	0.24	0.47	0.39	0.3	0.39	0.47	0.28	0.47	−0.41	0.24	0.4	1.0	−0.26	0.09
**WBI-5 Total**	−0.25	−0.27	−0.2	−0.16	−0.2	−0.28	−0.19	−0.17	0.1	−0.04	−0.42	−0.26	1.0	0.08
**BAS Total**	0.21	0.16	0.14	0.17	0.07	0.16	0.09	0.13	−0.04	0.07	0.31	0.09	0.08	1.0

**Note**: All values are Spearman’s rho, rounded to two decimal places. Variable abbreviations: see legend of Table 4.

### Construct validity of the Skindex-Mini

#### Correlational analyses.

As hypothesised for convergent validity (Table 5), the Skindex-Mini total score showed its strongest association with perceived stigma, as measured by the SSCI-8, *r*(547) = .426, *p* < .001), indicating a moderate positive relationship. Consistent with expectations, small but statistically significant positive correlations were found with the DERS-16 total score (*r* = .229, *p* < .001) and all of its subscales, including Non-acceptance of emotional responses (*r* = .193, *p* < .001), Difficulties engaging in goal-directed behaviour (*r* = .164, *p* < .001), Impulse control difficulties (*r* = .150, *p* < .001), Limited access to emotion regulation strategies (*r* = .212, *p* < .001), and Lack of emotional clarity (*r* = .246, *p* < .001).

The Skindex-Mini also correlated positively with distress as measured by the DH Total (*r*(135) = .293, *p* < .001) and with depressive symptoms assessed by the BDI-FS (*r*(139) = .236, *p* < .001), both in the small-to-moderate range. In line with the divergent validity hypothesis, no statistically significant correlation was observed with the, Anger-Out subscale of the AX Scale (*r*(513) = − .002, *p* = .963). Finally, as predicted for discriminant validity, a weak negative association was found with subjective well-being as measured by the WHO-5 (*r*(567) = − .247, *p* < .001), and no substantial correlation emerged with the BAS (*r*(544) = .209, *p* < .001), which was included as an unrelated construct.

Overall, the correlation pattern supports the hypothesised construct validity structure, with the Skindex-Mini demonstrating moderate associations with theoretically related constructs and negligible relationships with unrelated constructs.

#### Hierarchical regression analysis.

A hierarchical multiple regression analysis was conducted to examine the unique associations of demographic and psychosocial variables with Skindex-Mini scores, while controlling for the effects of all other predictors. Predictor variables were identical to those described in the Methods section and previously included in the correlation analysis. In Block 1, age and sex were entered as control variables. In Block 2, the psychometric variables were added.

The demographic block did not significantly predict Skindex-Mini scores (p > .05). The addition of the psychometric block significantly improved model fit (F(14, 113) = 3.587, p < .001), with an adjusted R² of 0.222, indicating a small effect size. However, it should be noted that the final regression model included only 128 cases after listwise deletion, yielding a cases-to-predictors ratio of approximately 9:1. This falls below the commonly recommended threshold of 10:1–15:1 for stable regression estimates, and consequently the analysis may be underpowered to detect smaller effects. Findings from the regression should therefore be interpreted as exploratory and hypothesis-generating rather than confirmatory.

In the final model, three predictors were statistically significant. Perceived stigma (SSCI-8) showed the strongest positive association (β = .354, *p* < .001). Anger suppression (HD Anger In) was also positively associated (β = .190, *p* = .029). In contrast, psychological well-being (WBI-5) demonstrated a negative association (β = –.323, *p* < .001).

Model assumptions were met: Variance Inflation Factor values ranged from 1.201 to 3.871, indicating no problematic multicollinearity; Cook’s distance values were < 1, suggesting the absence of influential outliers; residuals were independent (Durbin–Watson = 1.990, *p* = .930), homoscedastic, and approximately normally distributed.

These findings indicate that in this cross-sectional sample, higher perceived stigma and greater anger suppression were associated with higher Skindex-Mini scores, whereas higher psychological well-being was associated with lower Skindex-Mini scores, after adjusting for all other included variables.

#### Known-groups validity: Kruskal–Wallis analysis.

Known-groups validity was examined by comparing Skindex-Mini scores across the five DLQI-R categories (Table 6). Due to unmet parametric assumptions, a Kruskal–Wallis test was used. The analysis showed a significant difference across groups, $\chi^2(4) = 245.00$, $p < .001$, with a large effect size ($\epsilon^2 = .438$), indicating substantial discrimination (Table 6). Post-hoc comparisons confirmed that all DLQI-R categories scored significantly higher than the “no effect” group. Small: $W = 5.33, p = .002, r = 0.36$ (moderate effect), Moderate: $W = 9.09, p < .001, r = 0.50$ (large effect), Very large: $W = 11.04, p < .001, r = 0.57$ (large effect), Extremely large: $W = 11.69, p < .001, r = 0.62$ (large effect). This pattern of increasing scores confirms the scale’s ability to distinguish between clinically meaningful severity levels.

**Table 6:** Skindex-Mini scores across DLQI-R severity categories.

DLQI-R category	n	Mean	Median	SD	Min	Max
**No effect**	38	4.84	4	4.90	0	18
**Small effect**	120	7.83	8	4.27	0	18
**Moderate effect**	157	11.07	12	4.17	0	18
**Very large effect**	170	13.76	15	3.76	0	18
**Extremely large effect**	76	16.95	18	2.08	4	18

Values are presented as mean, median, standard deviation, minimum, and maximum. Abbreviations: DLQI-R, Dermatology Life Quality Index–Revised; SD, standard deviation.

#### Receiver operating characteristic analysis and optimal cutpoints for Skindex-Mini and subscales.

Based on the diagnostic performance indicators, a cut-off score of 13 is recommended for the Skindex-Mini total score. This threshold provides the optimal trade-off between sensitivity (76.83%) and specificity (73.97%), and yields the highest Youden’s index (0.508), indicating the most balanced diagnostic accuracy. Diagnostic Performance of the Skindex-Mini and its Items is presented in Table 7 Furthermore, this cutpoint maintains a high negative predictive value (80.34%) while preserving acceptable levels of positive predictive value (69.74%). The AUC (Area Under the Curve – value: .825) value indicates how accurately the Skindex-Mini scale can distinguish between clinical and non-clinical cases. An AUC of .825 reflects good discriminative ability. The Youden’s index is a summary measure that captures the performance of a diagnostic test by balancing sensitivity and specificity. In this case, the maximum Youden’s index is 0.508, which indicates a moderate overall accuracy of the Skindex-Mini scale in distinguishing between clinical and non-clinical groups. Therefore, a score of 13 or higher may serve as a clinically meaningful threshold for identifying individuals with significant dermatological QoL impairment.

**Table 7:** Diagnostic Performance of the Skindex-Mini and its Items.

Variable	Cutpoint	Sensitivity (%)	Specificity (%)	PPV (%)	NPV (%)	Youden’s index
**SM_1**	5	79.67	62.54	62.42	79.76	0.42
**SM_1**	6	65.45	76.83	68.8	74.01	0.42
**SM_2**	4	83.33	48.89	56.01	78.97	0.32
**SM_2**	5	71.95	63.49	60.62	74.35	0.35
**SM_2**	6	53.25	77.78	65.17	68.06	0.31
**SM_3**	3	87.8	57.14	61.54	85.71	0.45
**SM_3**	4	79.27	69.21	66.78	81.04	0.49
**SM_3**	5	63.82	84.44	76.21	74.93	0.48
**Skindex-Mini total**	12	81.71	65.08	64.63	82.0	0.47
**Skindex-Mini total**	13	76.83	73.97	69.74	80.34	0.51
**Skindex-Mini total**	14	71.54	78.73	72.43	77.99	0.5
**Skindex-Mini total**	15	63.41	84.44	76.1	74.72	0.48

**Fig 1:**
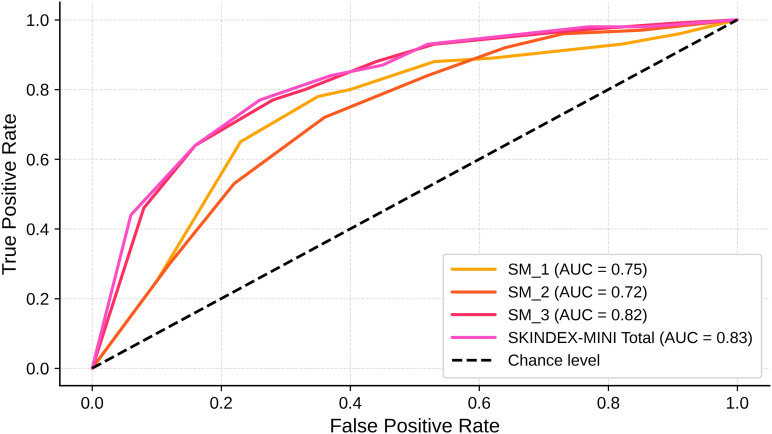
ROC Curves: Skindex-Mini items and total score. The curves illustrate the diagnostic performance of the individual items (SM1, SM2, SM3) and the total score. The diagonal line represents chance level performance.

The diagnostic performance of three item scales (SM1, SM2, and SM3) was examined using Receiver Operating Characteristic (ROC) analysis. To determine the optimal cutpoints, several indicators were considered, including sensitivity, specificity, positive and negative predictive values (PPV and NPV), Youden’s index, and the area under the ROC curve (AUC). Fig 1 presents the ROC Curves for the Skindex-Mini items and total score.

For SM1, a comparison of cutpoints 5 and 6 revealed similar AUC values (0.746) and Youden’s indices (0.422 and 0.423, respectively). However, the cutpoint of 6 yielded higher specificity (76.83%) and a more favorable positive predictive value (68.80%), despite a moderate decrease in sensitivity (65.45%). Thus, the cutpoint of 6 was deemed preferable.

In the case of SM2, three cutpoints were assessed. The value of 5 offered the most balanced trade-off between sensitivity (71.95%) and specificity (63.49%), accompanied by the highest Youden’s index (0.354). In contrast, the indices associated with cutpoints 4 and 6 were lower (0.322 and 0.310, respectively).

SM3 demonstrated the highest discriminative capacity, with an AUC of 0.818 across all evaluated cutpoints. The highest Youden’s index (0.485) was observed at cutpoint 4, which also displayed adequate sensitivity (79.27%) and specificity (69.21%). Although cutpoint 5 resulted in a slightly higher PPV (76.21%), it was accompanied by a considerable drop in sensitivity (63.82%), making it a less favorable trade-off overall.

In summary, the optimal cutpoints based on the ROC analysis were as follows: 6 for SM1, 5 for SM2, and 4 for SM3. These values offer the most effective balance between sensitivity and specificity, thereby maximizing the diagnostic utility of the scales.

The diagnostic performance of the Skindex-Mini at Optimal Cutpoints for Various Skin Disease Categories are presented in Table 8.

**Table 8:** Diagnostic Performance of the Skindex-Mini at Optimal Cutpoints for Various Skin Disease Categories.

	Cutpoint	Sensitivity (%)	Specificity (%)	AUC	PPV (%)	NPV (%)	Youden’s index	n
**1. Eczematous skin disorders: atopic dermatoses, contact dermatitis, dyshidrotic ekzema**	13	83.3%	74.2%	78.7%	68.50%	86.80%	0.57	149
**2. Sebaceous gland disorders: rosacea, acne**	13	73.9%	74.0%	73.9%	47.20%	90.00%	0.48	96
**3. Benign skin lesions: seborrhoeic keratoses**	15	66.7%	91.7%	79.2%	80.00%	84.60%	0.58	36
**4. Skin infections: warts**	16	62.5%	100.0%	81.3%	100.00%	66.70%	0.63	14
**5. Other: nail-disorder, non-scaring alopecia**	15	66.1%	88.1%	77.1%	84.80%	72.20%	0.54	118
**6. Allergic skin reactions: urticaria**	15	83.3%	75.0%	79.2%	66.70%	88.20%	0.58	32
**7. Autoimmune disorders: vitiligo**	12	78.6%	83.3%	81.0%	61.10%	92.10%	0.62	56
**8. Inflammatory disorders**	13	83.9%	71.1%	77.5%	77.70%	78.60%	0.55	251

#### Accessing the discriminatory capacity of the Skindex across dermatological diagnoses.

Sensitivity ranged from 62.5% (warts) to 83.9% (inflammatory disorders), and specificity from 66.7% to 100.0%. AUC was generally high, peaking at 81.3% for infectious disorders. While PPV varied—highest for warts (100.0%) and lowest for rosacea/acne (47.2%)—NPV was consistently high, reaching 92.1% for autoimmune conditions. Youden’s index (0.48–0.63) indicates moderately strong discriminatory power, particularly for infectious and autoimmune conditions.

Youden’s index, which summarizes overall test accuracy, ranged from 0.48 (sebaceous gland disorders) to 0.63 (warts), suggesting that the Skindex offers moderately strong discriminatory power, particularly in infectious and autoimmune conditions.

The analysis suggests that the Skindex scale shows weak to moderate ability to differentiate levels of quality-of-life impairment across various dermatological conditions. While predictive accuracy varied by diagnostic category, the scale performed particularly well in cases of infectious and autoimmune skin diseases. Overall, high NPV values across groups suggest that the Skindex is especially reliable in ruling out significant quality-of-life impairment when scores are low.

Notably, SM3 alone demonstrated an AUC (.818) comparable to that of the full Skindex-Mini and approaching the discriminatory performance reported for the DLQI-R in prior validation studies [8]. This finding highlights that even a single well-performing item may capture the core construct of dermatology-specific quality-of-life impairment with diagnostic accuracy similar to that of longer, established instruments, offering potential for ultra-brief screening applications in high-throughput clinical settings.

## Discussion

This study presents the first comprehensive evaluation of the Hungarian Skindex-Mini, confirming its robust reliability, validity, and utility for assessing health-related quality of life (HRQoL) in dermatological patients. Confirmatory factor analysis supported a single-factor structure, which is consistent with the design of the Mini as an ultra-brief indicator of dermatology-related burden derived from the multi-domain Skindex-16, although its three-item format necessarily limits the breadth of HRQoL content covered. Our results aligns with international validations in German, Italian, Spanish, and Chinese populations [9,10]. The Hungarian version performs similarly to these adaptations in terms of internal consistency and overall validity, while the nuanced findings discussed below may reflect sample-specific characteristics (e.g., diagnostic composition) or cultural factors influencing the reporting of HRQoL.

However, the item-level analysis provided further nuance beyond simple structural confirmation. Item Response Theory (IRT) analysis showed that all three items possess strong discriminatory power, effectively differentiating individuals across the spectrum of quality-of-life impairment. The scale appears most sensitive to capturing lower-to-mid levels of dermatology-related burden, particularly in relation to emotional and interpersonal impact, which may represent a critical window for early psychosocial intervention. While the three-item structure performed well, the IRT findings indicate that measurement precision diminishes at the most severe end of the impairment spectrum, likely because the three items cannot capture the full complexity and specific drivers of distress in patients with the most profound psychosocial involvement, where a more comprehensive instrument like the full Skindex-29 might be warranted for in-depth assessment.

A particularly noteworthy finding was the superior discriminative ability of item SM3, which assesses the impact of the skin condition on daily activities, such as going out, pursuing goals, or interacting with others. Its performance, with an area under the curve (AUC) of .818, approached that of the full Skindex-Mini scale and even comparable to established longer instruments like the DLQI-R [8]. This item’s high discriminative power likely stems from its capture of the profound interpersonal consequences of dermatological conditions. By asking about difficulty in “interacting with others,” SM3 taps directly into the social burden of skin diseases—the fear of negative evaluation, embarrassment, and subsequent social withdrawal that are particularly salient for patients with conditions affecting visible areas like the face, hands, or scalp. This finding strongly aligns with previous research highlighting stigmatization and social anxiety as primary drivers of poor quality of life in dermatology patients [1,29,30], reinforcing that the psychosocial impact, not just physical symptoms, defines the patient’s experience.

Construct validity was supported against broader measures than prior Skindex-16 work [31]. Regression analysis identified stigma, suppressed anger, and low well-being as independent predictors of HRQoL impairment. This aligns with evidence that stigma [1,29,30]—potentially outweighing clinical severity [29]—and anger [32,33] drive quality-of-life impairment. Notably, anger is a domain assessed in the Skindex-29 [34] and represents a potential target for psychological intervention [33], while the observed links with well-being correspond with the broader distress literature in dermatology [35,36]. Known-groups validity was further confirmed, as Skindex-Mini scores increased significantly and discriminatively across the DLQI-R’s established severity bands (none, mild, moderate, severe) [7,8]. This first comparison of the Skindex-Mini to the DLQI-R showed good diagnostic accuracy. While this convergence aligns with validations of longer Skindex versions [37–40], Skindex instruments may better capture the full breadth of emotional and functional domains compared to some other measures [38,41–44]. The Skindex-Mini identifies impairment comparably to longer instruments, though the specific clinical or research context should dictate instrument choice [41,42,44]. Importantly, performance was consistent across diagnostic subgroups, mirroring findings with longer versions [12,31,39–41,44].

### Clinical implications

The robust psychometric properties of the Hungarian Skindex-Mini, combined with its three-item format, have significant practical implications for routine clinical care. Its ultra-brief nature makes it exceptionally well-suited for time-constrained settings where longer questionnaires are impractical, including busy outpatient clinics, primary care practices, and teledermatology consultations. It can be administered, scored, and interpreted in under a minute, allowing for routine quality-of-life screening during every patient visit without disrupting clinic workflow.

The establishment of cut-off scores enhances the clinical utility of the instrument. Based on our analysis, a Skindex-Mini score of ≥13 (corresponding to the 75th percentile) represents a clinically meaningful threshold for identifying patients experiencing significant psychosocial burden. In practice, this cut-off can function as a “red flag,” prompting clinicians to initiate further discussion about the emotional and social impact of the skin condition, explore the need for psychosocial support, or consider referral to a psychodermatology specialist. Conversely, the high negative predictive value of the instrument means that scores below this threshold can reliably reassure clinicians that significant HRQoL impairment is unlikely, allowing consultation time to be focused on other aspects of care.

Beyond screening, the Skindex-Mini can inform treatment decisions and monitor response to interventions over time. By providing a standardized patient-reported outcome measure that captures the subjective burden of skin disease, the instrument complements objective clinical assessments (e.g., physician-rated severity scores) that may not fully reflect the patient’s lived experience. For instance, a patient with objectively mild psoriasis but a Skindex-Mini score above the clinical threshold may warrant more aggressive treatment or adjunctive psychological support than disease severity alone would suggest. Conversely, a patient whose clinical severity remains stable but whose Skindex-Mini score shows sustained improvement following an intervention provides valuable evidence of treatment effectiveness from the patient’s perspective. When administered longitudinally—such as at each follow-up visit—the Skindex-Mini can track changes in HRQoL over time, offering a sensitive measure of response to pharmacological, surgical, or psychological interventions.

The instrument’s brevity and simplicity also enhance its utility in emerging healthcare delivery models. In teledermatology, where non-verbal cues may be less accessible to clinicians, the Skindex-Mini can be easily integrated into electronic intake forms or sent to patients prior to virtual consultations, ensuring that the patient’s perspective on their HRQoL is systematically captured. Similarly, in primary care settings—where many dermatological conditions are initially managed but dermatology expertise may be limited—the Skindex-Mini provides a practical tool for identifying patients whose quality-of-life impairment warrants specialist referral. By making standardized QoL assessment feasible across diverse healthcare contexts, the Skindex-Mini can bridge the gap between recognizing the psychosocial burden of skin disease and actively monitoring and addressing it in clinical care.

### Limitations and future directions

The findings of this study should be interpreted in light of several limitations, which also point toward important directions for future research. First, the cross-sectional design precludes analysis of the Skindex-Mini’s responsiveness to change—a key attribute for a tool intended to monitor treatment efficacy over time. Longitudinal studies are urgently needed to establish the instrument’s sensitivity in detecting clinically meaningful improvements following pharmacological, surgical, or psychological interventions. Such studies would also enable the determination of minimal clinically important differences (MCID), further enhancing the utility of the Skindex-Mini as an outcome measure in both clinical practice and interventional research.

Second, the sample was characterized by a high proportion of female respondents (81%), which may affect generalizability. Research consistently indicates that women tend to report higher HRQoL impairment and may differ in emotion regulation patterns and illness perception compared to men; thus, the psychometric performance observed here should be confirmed in more gender-balanced cohorts. Future validation studies should also prioritize recruitment of more diverse clinical populations, including community-based samples, patients managed exclusively in primary care settings (who may have milder disease), and those requiring hospitalization for severe dermatological conditions. Multi-center studies incorporating these diverse care settings would substantially strengthen the generalizability of our findings.

Third, as identified through our IRT analysis, the Skindex-Mini demonstrates reduced measurement precision at higher levels of impairment. This suggests that while the instrument is excellent for screening and detecting mild-to-moderate HRQoL burden, it may be less sensitive to changes among patients with severe impairment—an important consideration when using the scale to monitor treatment response in severely affected populations. Although the three items cover symptom, emotional, and activity-related aspects of burden, the ultra-brief format necessarily limits content breadth. Accordingly, the Skindex-Mini should not be interpreted as a fully comprehensive representation of dermatology-specific HRQoL; rather, it may be best understood as a brief indicator of dermatology-related burden, with particular sensitivity to emotional and interpersonal impairment. Future research might explore whether this limitation could be addressed through the addition of one or two carefully selected items designed to capture the specific experiences of patients with profound HRQoL impairment, while maintaining the ultra-brief nature of the instrument. A modular approach could be considered, wherein the core three-item Skindex-Mini is administered universally, with optional follow-up items triggered only for patients who screen positive, allowing for more detailed assessment without burdening all patients.

Fourth, the hierarchical regression analysis was limited by a reduced effective sample size (N = 128 after listwise deletion) relative to the number of predictors (k = 14), resulting in a cases-to-predictors ratio of approximately 9:1. This raises concerns about statistical power and the stability of coefficient estimates. While multicollinearity was within acceptable limits (VIF range = 1.201–3.871) and model diagnostics were satisfactory, the risk of Type II errors cannot be excluded. Future studies with larger samples and lower rates of missing data are needed to confirm the independent predictors identified here.

Finally, while the high negative predictive value of the Skindex-Mini makes it suitable for rapid clinical screening, its performance in multinational research contexts should continue to be evaluated to ensure cross-cultural comparability. Comparative studies across different linguistic and cultural versions of the Skindex-Mini would help establish whether the instrument functions equivalently across populations, enabling its use in international trials and cross-cultural epidemiological research.

In conclusion, the Hungarian Skindex-Mini is a psychometrically robust, ultra-brief instrument that provides a valid and reliable indicator of dermatology-related burden, although its brevity necessarily limits the breadth of HRQoL content captured. Its strong performance, combined with its exceptional feasibility, positions it as a valuable tool for both clinical practice and research, capable of bringing the patient’s voice into routine dermatologic care.

## Statements and declarations

### Consent to participate

Written informed consent was obtained from all individual participants included in the study.

### Consent to publish

The authors affirm that human research participants provided informed consent for the publication of the data included in this article.

## Supporting information

S1 File Skindex-Mini QoL Health Questionnaire. (DOCX)

S2 File Dermatology Quality Index Revised (DLQI-R). (DOCX)

S3 File Difficulties in Emotion Regulation Scale (DERS-16). (DOCX)

S4 File SSCI-8 Questionnaire. (DOCX)

S5 File Visual Analogue Scale (EQ-VAS). (DOCX)

S6 File WHO-5 Well-Being Index. (DOCX)

S7 File Body Absorption Scale (BS). (DOCX)

S8 File Anger expression Scale (AX Scale). (DOCX)

S9 File Distress Thermometer (DH). (DOCX)

S10 File 9 item Beck Depression Inventory. (DOCX)
