# Supplementary material for: Reliability and construct validity of the Hungarian version of Skindex-Mini
Source: PLoS One. 2026 Jun 23;21(6):e0350749. doi: 10.1371/journal.pone.0350749 (PMC13289942; doi:10.1371/journal.pone.0350749)
Supplement: S1 File — (DOCX) [file pone.0350749.s001.docx]

**S1 Appendix Skindex-Mini QoL Health Questionnaire** (Skindex-Mini; Német et al., 2023; Swerlick et al., 2021

Skindex-Mini is an adapted version of the American instrument that has been proven reliable (Swerlick et al., 2021). Sociodemographic features included sex, age, diagnosed skin disease, and disease duration. The questionnaire was administered via Qualtrics (https://qualtrics.com) and implemented at the Department of Dermatology, Venereology, and Dermatooncology, Semmelweis University, Budapest, Hungary. In the clinical sample, dermatologists provided the diagnostic interview, and patients filled out the self-administered questionnaires in Qualtrics with the help of a trained psychology student.

It is a brief, dermatology-specific QoL measure comprising three items adapted from the Skindex-16, each rated on a 0–6 Likert-type scale, yielding a total score ranging from 0 to 18, with higher scores indicating greater QoL impairment. This abbreviated tool was developed to efficiently quantify the multidimensional burden of dermatological conditions, encompassing symptom severity, emotional distress, and functional impairment. Designed for clinical and research utility, the Skindex-Mini addresses the need for a rapid yet psychometrically robust assessment of skin disease-related QoL (Orenstein et al., 2020; Swerlick et al., 2021). Internal consistency for the SKINDEX total score was Cronbach’s α=0.78.
