# Supplementary material for: Reliability and construct validity of the Hungarian version of Skindex-Mini
Source: PLoS One. 2026 Jun 23;21(6):e0350749. doi: 10.1371/journal.pone.0350749 (PMC13289942; doi:10.1371/journal.pone.0350749)
Supplement: S2 File — (DOCX) [file pone.0350749.s002.docx]

**S2 Appendix Dermatology Life Quality Index-Revised (DLQI-R)** (DLQI-R, Finlay & Khan, 1994; Rencz et al., 2020)

DLQI-R is a condition-specific QoL instrument that has been extensively validated for use across more than 40 dermatological conditions over its 25-year history. This revised version incorporates an alternative scoring system that demonstrates enhanced psychometric properties compared to the original DLQI. The questionnaire assesses ten domains of dermatology-specific QoL impairment, like symptom severity and discomfort, skin tightness, activities of daily living, clothing restrictions, social and leisure activities, sports participation, work or academic performance, interpersonal relationships, sexual functioning, treatment burden, and skin care challenges. The DLQI-R (Dermatology Life Quality Index – Revised) consists of 10 items rated from 0 (“not at all”) to 3 (“very much”), with an additional “not relevant” option coded as -99. These “not relevant” responses were treated as user-missing values in SPSS. The raw DLQI-R score was calculated as the sum of all valid responses. To account for missing values, the maximum possible score was adjusted to the number of valid items (valid items × 3). The final DLQI-R score was then rescaled to a 0–30 range using the formula: DLQI-R Score = (Raw Score / (Valid Items × 3)) × 30. The total score ranges from 0 (no QoL impairment) to 30 (maximum impairment). DLQI‑R total scores were classified into five validated impact bands—0–1 (no effect), 2–5 (small effect), 6–10 (moderate effect), 11–20 (very large effect), and 21–30 (extremely large effect)—using the established DLQI cut‑offs, as these bandings have been shown to remain valid when applied to DLQI‑R scores. with the following clinical interpretations. 0-10: Mild to moderate impact, 11-30: Severe QoL impairment (indicating substantial disease burden). DLQI‑R total scores were classified into five validated impact bands—0–1 (no effect), 2–5 (small effect), 6–10 (moderate effect), 11–20 (very large effect), and 21–30 (extremely large effect)—using the established DLQI cut‑offs, as these bandings have been shown to remain valid when applied to DLQI‑R scores. Receiver Operating Characteristic (ROC) curves were computed to evaluate the criterion validity of the SKINDEX-MINI scale and its individual items (SM_1, SM_2, SM_3) in detecting clinically significant dermatologic quality-of-life impairment. The binary classification criterion was based on DLQI-R categories 4 and 5 (DLQI-R > 10), reflecting moderate to very large impact. The DLQI-R maintains the original instrument's robust measurement properties while offering improved sensitivity to detect clinically meaningful changes in QoL (Rencz, Gulácsi, et al., 2020a, 2020b).
