# Supplementary material for: Reliability and construct validity of the Hungarian version of Skindex-Mini
Source: PLoS One. 2026 Jun 23;21(6):e0350749. doi: 10.1371/journal.pone.0350749 (PMC13289942; doi:10.1371/journal.pone.0350749)
Supplement: S3 File — (DOCX) [file pone.0350749.s003.docx]

**S3 Appendix Difficulties in Emotion Regulation Scale-16 (DERS-16)** (DERS-16; (Bjureberg et al., 2016; Kökönyei et al., 2014)

DERS-16 is a theoretically grounded, psychometrically validated 16-item abbreviated measure of emotion dysregulation. Derived from the original 36-item DERS, this shortened version maintains robust reliability and validity while improving clinical utility through reduced respondent burden. The scale has demonstrated strong measurement psychometric properties across a clinical sample (N = 96) and two large community samples (Ns = 102 and 482). The subscale structure of the DERS-16 assesses five theoretically derived dimensions of emotion dysregulation, which are the following (Bjureberg et al., 2016): Non-acceptance of emotional responses (α=0.84), Difficulties engaging in goal-directed behavior (α=0,81), Impulse control difficulties (α=0.89), Limited access to emotion regulation strategies (α=0·88), and Lack of emotional clarity (α=0·59), which lower α is expected for a 2-item scale. The DERS-16 total score showed excellent internal consistency (α=0·93).
