# Supplementary material for: Reliability and construct validity of the Hungarian version of Skindex-Mini
Source: PLoS One. 2026 Jun 23;21(6):e0350749. doi: 10.1371/journal.pone.0350749 (PMC13289942; doi:10.1371/journal.pone.0350749)
Supplement: S4 File — (DOCX) [file pone.0350749.s004.docx]

**S4 Appendix Stigmatization Scale for Chronic Illnesses–8 Questionnaire (SSCI-8)** (SSCI-8, Molina et al., 2013; Szőcs et al., 2021)

SSCI-8 is an 8-item unidimensional instrument designed to measure perceived and internalized stigma in individuals with chronic illnesses. Developed from a validated longer scale, the SSCI-8 demonstrates strong internal consistency and correlates with measures of psychological distress and functional impairment. Responses were recorded on a 5-point frequency scale (1 = Never; 2 = Rarely; 3 = Sometimes; 4 = Often; 5 = Always). The raw summed scores range from 8 to 40, with higher scores indicating greater stigma (Molina et al., 2013; Szőcs et al., 2021). Internal consistency for the SSCI-8 total score was Cronbach’s α=0·78.
