# Supplementary material for: Reliability and construct validity of the Hungarian version of Skindex-Mini
Source: PLoS One. 2026 Jun 23;21(6):e0350749. doi: 10.1371/journal.pone.0350749 (PMC13289942; doi:10.1371/journal.pone.0350749)
Supplement: S5 File — (DOCX) [file pone.0350749.s005.docx]

**S5 Appendix Visual Analog Scale (EQ-VAS)** (EQ-5D; Balestroni & Bertolotti, 2015; Rencz et al., 2022)

EQ-VAS is a standardized, self-administered instrument developed by the EuroQol Group to assess health-related QoL (HRQoL). Widely utilized in clinical and health economic research, it evaluates five dimensions: mobility, self-care, usual activities, pain/discomfort, and anxiety/depression. Each dimension is rated on a 3-level severity scale (no problems, some problems, extreme problems). The EQ-5D includes two components. Descriptive System: Provides a health state profile (e.g., "12123" indicating no mobility issues, some self-care difficulties, etc.). Visual Analog Scale (EQ-VAS): A quantitative measure where respondents rate their overall health from 0 ("worst imaginable health state") to 100 ("best imaginable health state"). This dual-approach design enables both qualitative and quantitative assessments of HRQoL, making it valuable for cost-effectiveness analyses and cross-population comparisons (Balestroni & Bertolotti, 2015). In our study, we only used the Visual Analog Scale (EQ-VAS).
