# Supplementary material for: Reliability and construct validity of the Hungarian version of Skindex-Mini
Source: PLoS One. 2026 Jun 23;21(6):e0350749. doi: 10.1371/journal.pone.0350749 (PMC13289942; doi:10.1371/journal.pone.0350749)
Supplement: S6 File — (DOCX) [file pone.0350749.s006.docx]

**S6 Appendix WHO-5 Well-Being Index (WBI-5)** (WBI-5; Susánszky et al., 2006; Topp et al., 2015)

The WHO-5 Well-Being Index (WBI-5) is a concise 5-item self-report questionnaire derived from the original WHO-5, designed to assess subjective psychological well-being over the preceding two weeks. This widely used tool demonstrates strong psychometric properties and cross-cultural validity (Topp et al., 2015). Items are rated on a 6-point Likert scale, recoded to 0–5 (5: All of the time; 4: Most of the time; 3: More than half of the time; 2: Less than half of the time; 1: Some of the time; 0: At no time). Scoring: Raw score: Sum of item scores (range: 0–25). Final score: Raw score × 4 (range: 0–100), where 0 = worst imaginable well-being and 100 = optimal well-being. Reliability: Excellent internal consistency (Cronbach’s α = 0.85) in the Hungarian validation study (Susánszky et al., 2006). Internal consistency for the WBI-5 total score was Cronbach’s α=.0.85.
