# Supplementary material for: Reliability and construct validity of the Hungarian version of Skindex-Mini
Source: PLoS One. 2026 Jun 23;21(6):e0350749. doi: 10.1371/journal.pone.0350749 (PMC13289942; doi:10.1371/journal.pone.0350749)
Supplement: S7 File — (DOCX) [file pone.0350749.s007.docx]

**S7 Appendix Body Absorption Scale** (BAS, Köteles et al., 2012)

BAS is a 19-item unidimensional instrument designed to assess sustained, non-evaluative attention to normative somatic processes (Watson, 2005). Developed to distinguish adaptive bodily awareness from pathological symptom monitoring, the BAS demonstrates robust psychometric properties and unique theoretical utility. The BAS comprises 17 positively keyed items and 2 reverse-scored items (Items 6, 10), rated on a 5-point Likert-type scale (1 = "Never" to 5 = "Always"). Summed scores (range: 19–95) reflect the intensity of bodily absorption, with higher scores indicating greater attentional focus. Notably, the scale lacks established clinical cutoffs; scores are interpreted dimensionally to quantify individual differences in non-pathological somatic monitoring. Confirmatory factor analysis supports a single-factor solution (CFI > 0.90, RMSEA < 0.06), confirming the scale’s unidimensionality (Köteles et al., 2012). The BAS exhibits strong internal consistency (α = 0.84–0.91) and test-retest reliability (ICC = 0.78 over 2 weeks). Convergent validity correlates moderately with neuroticism (r = 0.32–0.41) and inversely with mindfulness (r = −0.25). Discriminant validity has minimal association with health anxiety (r < 0.20), underscoring its focus on normative (vs. pathological) processes (Köteles et al., 2012; Watson, 2005). The internal consistency for the BAS total score was Cronbach’s α = 0.87.

**S4 Appendix Stigmatization Scale for Chronic Illnesses–8 Questionnaire (SSCI-8)** (SSCI-8, Molina et al., 2013; Szőcs et al., 2021)

SSCI-8 is an 8-item unidimensional instrument designed to measure perceived and internalized stigma in individuals with chronic illnesses. Developed from a validated longer scale, the SSCI-8 demonstrates strong internal consistency and correlates with measures of psychological distress and functional impairment. Responses were recorded on a 5-point frequency scale (1 = Never; 2 = Rarely; 3 = Sometimes; 4 = Often; 5 = Always). The raw summed scores range from 8 to 40, with higher scores indicating greater stigma (Molina et al., 2013; Szőcs et al., 2021). Internal consistency for the SSCI-8 total score was Cronbach’s α=0·78.

**S5 Appendix Visual Analog Scale (EQ-VAS)** (EQ-5D; Balestroni & Bertolotti, 2015; Rencz et al., 2022)

EQ-VAS is a standardized, self-administered instrument developed by the EuroQol Group to assess health-related QoL (HRQoL). Widely utilized in clinical and health economic research, it evaluates five dimensions: mobility, self-care, usual activities, pain/discomfort, and anxiety/depression. Each dimension is rated on a 3-level severity scale (no problems, some problems, extreme problems). The EQ-5D includes two components. Descriptive System: Provides a health state profile (e.g., "12123" indicating no mobility issues, some self-care difficulties, etc.). Visual Analog Scale (EQ-VAS): A quantitative measure where respondents rate their overall health from 0 ("worst imaginable health state") to 100 ("best imaginable health state"). This dual-approach design enables both qualitative and quantitative assessments of HRQoL, making it valuable for cost-effectiveness analyses and cross-population comparisons (Balestroni & Bertolotti, 2015). In our study, we only used the Visual Analog Scale (EQ-VAS).

**S6 Appendix WHO-5 Well-Being Index (WBI-5)** (WBI-5; Susánszky et al., 2006; Topp et al., 2015)

The WHO-5 Well-Being Index (WBI-5) is a concise 5-item self-report questionnaire derived from the original WHO-5, designed to assess subjective psychological well-being over the preceding two weeks. This widely used tool demonstrates strong psychometric properties and cross-cultural validity (Topp et al., 2015). Items are rated on a 6-point Likert scale, recoded to 0–5 (5: All of the time; 4: Most of the time; 3: More than half of the time; 2: Less than half of the time; 1: Some of the time; 0: At no time). Scoring: Raw score: Sum of item scores (range: 0–25). Final score: Raw score × 4 (range: 0–100), where 0 = worst imaginable well-being and 100 = optimal well-being. Reliability: Excellent internal consistency (Cronbach’s α = 0.85) in the Hungarian validation study (Susánszky et al., 2006). Internal consistency for the WBI-5 total score was Cronbach’s α=.0.85.

**S7 Appendix Body Absorption Scale (BAS)** (BAS, Köteles et al., 2012)

BAS is a 19-item unidimensional instrument designed to assess sustained, non-evaluative attention to normative somatic processes (Watson, 2005). Developed to distinguish adaptive bodily awareness from pathological symptom monitoring, the BAS demonstrates robust psychometric properties and unique theoretical utility. The BAS comprises 17 positively keyed items and 2 reverse-scored items (Items 6, 10), rated on a 5-point Likert-type scale (1 = "Never" to 5 = "Always"). Summed scores (range: 19–95) reflect the intensity of bodily absorption, with higher scores indicating greater attentional focus. Notably, the scale lacks established clinical cutoffs; scores are interpreted dimensionally to quantify individual differences in non-pathological somatic monitoring. Confirmatory factor analysis supports a single-factor solution (CFI > 0.90, RMSEA < 0.06), confirming the scale’s unidimensionality (Köteles et al., 2012). The BAS exhibits strong internal consistency (α = 0.84–0.91) and test-retest reliability (ICC = 0.78 over 2 weeks). Convergent validity correlates moderately with neuroticism (r = 0.32–0.41) and inversely with mindfulness (r = −0.25). Discriminant validity has minimal association with health anxiety (r < 0.20), underscoring its focus on normative (vs. pathological) processes (Köteles et al., 2012; Watson, 2005). The internal consistency for the BAS total score was Cronbach’s α = 0.87.

**S8 Appendix Anger Expression Scale (AX Scale)** (AX Scale, Spielberger et al., 1985, Knight et al., 1988; Supplementary material G)

Anger Expression Scale (AX Scale, Spielberger et al., 1985, Knight et al., 1988) is a 20-item self-report questionnaire designed to assess individual differences in the expression and suppression o f anger as a stable personality trait. The Hungarian adaptation was validated by Oláh Attila (Csibi et al., 2010). The subscales of the AX Scale are: Anger Expression (A/EX): Frequency of anger expression (sum score range: 20–80); Anger-In (A/I): Tendency to suppress anger (range: 8–32); Anger-Out (A/O): Tendency to outwardly vent anger (range: 8–32). The response format of the AX Scale is so that the items are rated on a 4-point Likert scale, assessing how often respondents engage in specific anger-related behaviors. AX Scale has a good internal consistency (Cronbach’s α = 0.75) in Hungarian samples (Csibi et al., 2010), and effectively discriminates between anger expression styles (Knight et al., 1988). Internal consistency for the Anger-In and Anger-Out subscales was Cronbach’s α = 0.71 and 0.84, respectively.

**S9 Appendix Distress Thermometer (DT)** (Mailáth et al., 2017; Roth et al., 1998; (DT)

DT is a rapid screening tool for psychological distress that demonstrates strong psychometric properties across various clinical populations. Originally developed for oncology settings, it has since been validated for broader use in primary care (Mailáth et al., 2017; Roth et al., 1998).
