# Supplementary material for: Reliability and construct validity of the Hungarian version of Skindex-Mini
Source: PLoS One. 2026 Jun 23;21(6):e0350749. doi: 10.1371/journal.pone.0350749 (PMC13289942; doi:10.1371/journal.pone.0350749)
Supplement: S8 File — (DOCX) [file pone.0350749.s008.docx]

**S8 Appendix Anger Expression Scale (AX Scale)** (AX Scale, Spielberger et al., 1985, Knight et al., 1988; Supplementary material G)

Anger Expression Scale (AX Scale, Spielberger et al., 1985, Knight et al., 1988) is a 20-item self-report questionnaire designed to assess individual differences in the expression and suppression o f anger as a stable personality trait. The Hungarian adaptation was validated by Oláh Attila (Csibi et al., 2010). The subscales of the AX Scale are: Anger Expression (A/EX): Frequency of anger expression (sum score range: 20–80); Anger-In (A/I): Tendency to suppress anger (range: 8–32); Anger-Out (A/O): Tendency to outwardly vent anger (range: 8–32). The response format of the AX Scale is so that the items are rated on a 4-point Likert scale, assessing how often respondents engage in specific anger-related behaviors. AX Scale has a good internal consistency (Cronbach’s α = 0.75) in Hungarian samples (Csibi et al., 2010), and effectively discriminates between anger expression styles (Knight et al., 1988). Internal consistency for the Anger-In and Anger-Out subscales was Cronbach’s α = 0.71 and 0.84, respectively.
