# Supplementary material for: Reliability and construct validity of the Hungarian version of Skindex-Mini
Source: PLoS One. 2026 Jun 23;21(6):e0350749. doi: 10.1371/journal.pone.0350749 (PMC13289942; doi:10.1371/journal.pone.0350749)
Supplement: S9 File — (DOCX) [file pone.0350749.s009.docx]

**S9 Appendix Distress Thermometer (DT)** (Mailáth et al., 2017; Roth et al., 1998; (DT)

DT is a rapid screening tool for psychological distress that demonstrates strong psychometric properties across various clinical populations. Originally developed for oncology settings, it has since been validated for broader use in primary care (Mailáth et al., 2017; Roth et al., 1998).
