# Supplementary material for: Reliability and construct validity of the Hungarian version of Skindex-Mini
Source: PLoS One. 2026 Jun 23;21(6):e0350749. doi: 10.1371/journal.pone.0350749 (PMC13289942; doi:10.1371/journal.pone.0350749)
Supplement: S10 File — (DOCX) [file pone.0350749.s010.docx]

**S10 Appendix 9-item Beck Depression Inventory (BDI-FS)** (Furlanetto et al., 2005; Rózsa et al., 2001; BDI-FS).

BDI-FS is a shortened, unidimensional version of the original BDI-II, designed to screen for depressive symptoms with high diagnostic accuracy. Scoring and interpretation of the Scale are as follows: 0–9 Normal range; 10–18 Mild depression; 19–25 Moderate depression; ≥26 Severe depression. The scale’s reliability shows excellent internal consistency (Cronbach’s α > 0.90), and confirmatory factor analysis supports a unidimensional structure. The scale also shows high sensitivity and specificity (>90%) against DSM-III-R diagnoses (using DIS) at a cut-off score of 16. Respondents scoring ≥16 (indicating probable depression) receive feedback within two weeks, advising consultation with a psychologist or psychiatrist (Furlanetto et al., 2005; Rózsa et al., 2001). Internal consistency for the BDI-FS total score was Cronbach’s α=0·79.
