# Supplementary material for: Reliability and construct validity of the Hungarian version of Skindex-Mini
Source: PLoS One. 2026 Jun 23;21(6):e0350749. doi: 10.1371/journal.pone.0350749 (PMC13289942; doi:10.1371/journal.pone.0350749)
Supplement: Attachment — Submitted filename: Response to Reviewers.docx [file pone.0350749.s012.docx]

**Response to Reviewers**

**Manuscript ID:** PONE-D-26-03636
**Title:** Reliability and Construct Validity of the Hungarian Version of Skindex-Mini
**Journal:** PLOS One
**Corresponding Author:** Borbála Német

Dear Dr. Chernyshov and Reviewers,

We sincerely thank you for the opportunity to revise our manuscript entitled "Reliability and Construct Validity of the Hungarian Version of Skindex-Mini." We are grateful to the Academic Editor and the reviewers for their thoughtful and constructive comments, which have helped us substantially improve the quality of our work.

We have carefully addressed all points raised in the review process. Below, we provide a point-by-point response to each comment, describing the changes made to the manuscript. All modifications have been marked with track changes in the revised manuscript file.

**Response to Reviewer #1**

We thank Reviewer #1 for the positive assessment of our work and the constructive suggestions for improvement.

**Comment 1.1:** *"The Discussion section could benefit from a more in-depth interpretation of the findings. Specifically, the authors could elaborate on why certain items (e.g., SM3) demonstrate high discriminative ability and discuss the practical implications for ultra-brief screening in clinical settings."*

**Response:** We agree with this valuable suggestion. We have expanded the Discussion section to include a more detailed interpretation of item-level findings. Specifically, we have added a paragraph elaborating on why item SM3 ("Your skin condition made it difficult to show affection") demonstrated particularly high discriminative ability, noting that this item captures the interpersonal and emotional consequences of skin diseases that may be especially salient in conditions affecting visible areas. We also discuss how this finding aligns with previous research highlighting the social burden of dermatological conditions. Additionally, we have elaborated on the practical implications of the Skindex-Mini as an ultra-brief screening tool, emphasizing its utility in time-constrained clinical settings, its potential for routine use to identify patients in need of psychosocial support, and its ease of administration in both outpatient and teledermatology contexts.

**Location of change:** *We have rewritten the Discussion section*.

**Comment 1.2:** *"Although the Introduction provides a detailed comparison with previous validation studies across languages and conditions, the Discussion does not fully contextualize the present findings within this broader literature, such as similarities or differences in performance across dermatological populations."*

**Response:** We thank the reviewer for highlighting this important gap. We have substantially revised the Discussion to better contextualize our findings within the broader literature on Skindex-Mini validations. We now explicitly compare our psychometric findings (internal consistency, factor structure, convergent validity) with those reported in previous validation studies from other linguistic and cultural contexts (e.g., German, Italian, Spanish, Chinese versions). We discuss how the Hungarian version performs similarly to other adaptations in terms of reliability and validity, while noting that slight variations may be attributable to sample characteristics (e.g., diagnostic composition) or cultural factors in health-related quality of life reporting.

**Location of change:** *We have rewritten the Discussion section*.

**Comment 1.3:** *"Limitations should be mentioned, such as the high proportion of female respondents, potential recruitment bias, and reduced precision at higher levels of impairment."*

**Response:** We appreciate this important observation. We have significantly expanded the Limitations section to address these points explicitly. We now acknowledge the overrepresentation of female participants (81%) as a limitation that may affect generalizability, as women typically report higher HRQoL impairment and may differ in emotion regulation patterns. We also discuss potential recruitment bias stemming from the single-center, outpatient clinic setting, which may not represent the full spectrum of dermatological patients (e.g., those with milder conditions managed in primary care or severe cases requiring hospitalization). Additionally, we note the reduced precision of the Skindex-Mini at higher levels of impairment as identified through our IRT analysis, suggesting that the instrument may be less sensitive to changes among patients with severe HRQoL burden—an important consideration for monitoring treatment response in severely affected populations.

**Location of change:** *We have rewritten the Discussion section, Limitations subsection.*

*“****Limitations and Future Directions***

*Second, the sample was characterized by a high proportion of female respondents (81%), which may affect generalizability. Research consistently indicates that women tend to report higher HRQoL impairment and may differ in emotion regulation patterns and illness perception compared to men; thus, the psychometric performance observed here should be confirmed in more gender-balanced cohorts. Future validation studies should also prioritize recruitment of more diverse clinical populations, including community-based samples, patients managed exclusively in primary care settings (who may have milder disease), and those requiring hospitalization for severe dermatological conditions. Multi-center studies incorporating these diverse care settings would substantially strengthen the generalizability of our findings.”*

**Comment 1.4:** *"The authors could also expand on clinical implications, such as how the cut-off scores could be used in practice, how the Skindex-Mini could inform treatment decisions or monitor response to interventions, and the possible use in teledermatology or primary care."*

**Response:** We thank the reviewer for this practical focus. We have added a new subsection on "Clinical Implications" within the Discussion. In this section, we elaborate on how the established cut-off scores (using the 75th percentile as a threshold for clinically significant impairment) can be readily implemented in routine practice to identify patients who may benefit from additional psychosocial support or referral. We discuss how the Skindex-Mini can complement clinical assessment by providing a patient-reported outcome measure that captures the subjective burden of skin disease, potentially informing treatment decisions (e.g., escalation of therapy when HRQoL impairment is severe) and monitoring response to interventions over time. We also highlight the particular utility of this ultra-brief instrument in teledermatology consultations and busy primary care settings where time is limited, enabling systematic screening for HRQoL impairment that might otherwise be overlooked.

**Location of change:** *Discussion section, Clinical Implications subsection*

*“****Clinical Implications***

*The robust psychometric properties of the Hungarian Skindex-Mini, combined with its three-item format, have significant practical implications for routine clinical care. Its ultra-brief nature makes it exceptionally well-suited for time-constrained settings where longer questionnaires are impractical, including busy outpatient clinics, primary care practices, and teledermatology consultations. It can be administered, scored, and interpreted in under a minute, allowing for routine quality-of-life screening during every patient visit without disrupting clinic workflow.*

*The establishment of cut-off scores enhances the clinical utility of the instrument. Based on our analysis, a Skindex-Mini score of ≥13 (corresponding to the 75th percentile) represents a clinically meaningful threshold for identifying patients experiencing significant psychosocial burden. In practice, this cut-off can function as a "red flag," prompting clinicians to initiate further Discussion about the emotional and social impact of the skin condition, explore the need for psychosocial support, or consider referral to a psychodermatology specialist. Conversely, the high negative predictive value of the instrument means that scores below this threshold can reliably reassure clinicians that significant HRQoL impairment is unlikely, allowing consultation time to be focused on other aspects of care.*

*Beyond screening, the Skindex-Mini can inform treatment decisions and monitor response to interventions over time. By providing a standardized patient-reported outcome measure that captures the subjective burden of skin disease, the instrument complements objective clinical assessments (e.g., physician-rated severity scores) that may not fully reflect the patient's lived experience. For instance, a patient with objectively mild psoriasis but a Skindex-Mini score above the clinical threshold may warrant more aggressive treatment or adjunctive psychological support than disease severity alone would suggest. Conversely, a patient whose clinical severity remains stable but whose Skindex-Mini score shows sustained improvement following an intervention provides valuable evidence of treatment effectiveness from the patient's perspective. When administered longitudinally—such as at each follow-up visit—the Skindex-Mini can track changes in HRQoL over time, offering a sensitive measure of response to pharmacological, surgical, or psychological interventions.*

*The instrument's brevity and simplicity also enhance its utility in emerging healthcare delivery models. In teledermatology, where non-verbal cues may be less accessible to clinicians, the Skindex-Mini can be easily integrated into electronic intake forms or sent to patients prior to virtual consultations, ensuring that the patient's perspective on their HRQoL is systematically captured. Similarly, in primary care settings—where many dermatological conditions are initially managed but dermatology expertise may be limited—the Skindex-Mini provides a practical tool for identifying patients whose quality-of-life impairment warrants specialist referral. By making standardized QoL assessment feasible across diverse healthcare contexts, the Skindex-Mini can bridge the gap between recognizing the psychosocial burden of skin disease and actively monitoring and addressing it in clinical care.”*

**Comment 1.5:** *"They could suggest directions for future validation, e.g., longitudinal studies to assess responsiveness to change, studies in more diverse clinical populations, or adding items to improve measurement at severe impairment levels."*

**Response:** We agree and have expanded the Future Directions section accordingly. The revised Discussion now highlights the need for longitudinal studies examining responsiveness to change, validation in more diverse clinical populations, and possible strategies for improving measurement precision at the severe end of the burden spectrum while preserving the instrument’s brevity.

**Location of change:** *Discussion section, Limitations and Future Directions subsection.*

***“******Limitations and Future Directions***

*The findings of this study should be interpreted in light of several limitations, which also point toward important directions for future research. First, the cross-sectional design precludes analysis of the Skindex-Mini's responsiveness to change—a key attribute for a tool intended to monitor treatment efficacy over time. Longitudinal studies are urgently needed to establish the instrument's sensitivity in detecting clinically meaningful improvements following pharmacological, surgical, or psychological interventions. Such studies would also enable the determination of minimal clinically important differences (MCID), further enhancing the utility of the Skindex-Mini as an outcome measure in both clinical practice and interventional research.*

*…*

*Third, as identified through our IRT analysis, the Skindex-Mini demonstrates reduced measurement precision at higher levels of impairment. This suggests that while the instrument is excellent for screening and detecting mild-to-moderate HRQoL burden, it may be less sensitive to changes among patients with severe impairment—an important consideration when using the scale to monitor treatment response in severely affected populations. Future research might explore whether this limitation could be addressed through the addition of one or two carefully selected items designed to capture the specific experiences of patients with profound HRQoL impairment, while maintaining the ultra-brief nature of the instrument. A modular approach could be considered, wherein the core three-item Skindex-Mini is administered universally, with optional follow-up items triggered only for patients who screen positive, allowing for more detailed assessment without burdening all patients.*

*Finally, while the high negative predictive value of the Skindex-Mini makes it suitable for rapid clinical screening, its performance in multinational research contexts should continue to be evaluated to ensure cross-cultural comparability. Comparative studies across different linguistic and cultural versions of the Skindex-Mini would help establish whether the instrument functions equivalently across populations, enabling its use in international trials and cross-cultural epidemiological research.*

*In conclusion, the Hungarian Skindex-Mini is a psychometrically robust, ultra-brief instrument that provides a valid and reliable indicator of dermatology-related burden, although its brevity necessarily limits the breadth of HRQoL content captured. Its strong performance, combined with its exceptional feasibility, positions it as a valuable tool for both clinical practice and research, capable of bringing the patient's voice into routine dermatologic care.****”***

**Comment 1.6:** *"In the Methods it would be useful to include a brief description of the Skindex-Mini and its scoring."*

**Response:** We agree that this information is essential for readers unfamiliar with the instrument. We have added a brief description of the Skindex-Mini to the Methods section, Instruments subsection, including the number of items (3), the response scale (5-point Likert scale from "never" to "all the time"), the scoring method (summed raw scores converted to a 0-100 linear scale), and interpretation (higher scores indicate greater HRQoL impairment). We also note the domains covered (symptoms, emotions, social functioning) as represented by the three items.

**Location of change:** *Methods section, Instruments subsection*

## *“****Instruments***

*To assess the construct validity of the Hungarian Skindex-Mini, participants completed a series of established, validated questionnaires covering health-related quality of life, emotion regulation, stigma, and psychological well-being. For each instrument described below, the internal consistency (Cronbach's α) was calculated for the current sample and is reported to provide context for the subsequent analyses.*

***Dermatology-Specific Quality of Life***

- ***Skindex-Mini QoL Health Questionnaire (Skindex-Mini)*** *[9] is a 3-item ultrashort measure designed to assess dermatology-specific health-related quality of life (HRQoL). Each item represents a core domain: symptom burden (e.g., "In the past week, how often were you bothered by your skin symptoms...?"), emotional impact (e.g., "...how often did your skin symptoms cause emotional difficulties...?"), and functional limitations (e.g., "...how often did your skin symptoms make it difficult for you to engage in activities...?"). Respondents rate the frequency of each experience over the past week on a 7-point Likert scale (0 = Never to 6 = All the time). Item scores are summed to produce a total raw score (range 3--15), which is then transformed to a 0--100 linear scale, with higher scores indicating greater impairment (worse QoL). The internal consistency in the current sample was good (Cronbach's α = 0.78).”*

**Comment 1.7:** *"In the note of Table 4 it is written 'All correlations are Spearman's rho'. However, there are no correlations in the table."*

**Response:** We thank the reviewer for identifying this error. Table 4 presents regression results, not correlations. The note was incorrectly retained from an earlier version of the manuscript. We have removed this erroneous note from Table 4 and ensured that the table title and notes accurately reflect the content (hierarchical regression analysis).

**Location of change:** *Table 4, notes section*

*“****Note:*** *Variable abbreviations: Skindex-Mini = Skindex-Mini QoL Health Questionnaire; DERS-16 Total = Difficulties in Emotion Regulation Scale-16 total score; DERS-16 Non-Accept. = Non-acceptance of emotional responses; DERS-16 Goal. Dir. Behav. = Difficulties engaging in goal-directed behaviour; DERS-16 Imp. Contr. Diff. = Impulse control difficulties; DERS-16 Em. Regul. = Limited access to emotion regulation strategies; DERS-16 Lack. Em. Clarity = Lack of emotional clarity; SSCI-8 = Stigmatization Scale for Chronic Illnesses–8; HD Anger In = Anger-In subscale of the Anger Expression Scale; HD Anger Out = Anger-Out subscale of the Anger Expression Scale; DH Types Total = Distress Thermometer total score; BD Total = Beck Depression Inventory-Fast Screen total score; WBI-5 Total = WHO-5 Well-Being Index total score; BAS Total = Bodily Absorption Scale total score.”*

**Response to Reviewer #2 (Dr. Flora Balieva)**

We thank Dr. Balieva for the detailed and insightful review, which has helped us strengthen the methodological rigor and clarity of our manuscript.

**Comment 2.1:** *"Using KMO and Bartlett in a CFA framework is conceptually inconsistent."*

**Response:** Thank you for this important observation. We agree that the KMO measure and Bartlett’s test are traditionally used in the context of exploratory factor analysis rather than confirmatory factor analysis. Following the reviewer’s suggestion, we have removed these statistics from the manuscript and now focus exclusively on the CFA model fit indices when evaluating the factorial structure of the Skindex-Mini.

**Location of change:** Specifically, we removed the following sentence from the Results section: “*The Kaiser–Meyer–Olkin measure of sampling adequacy (KMO = 0.713) and Bartlett’s test of sphericity (χ²(3) = 706.270, p < 0.001) supported the suitability of the data for factor analysis.*”

**Comment 2.2:** *"IRT with 3 items is unstable and clinically limited. This is acknowledged as a limitation. I suggest you consider addressing whether the scale is functionally an 'emotional impairment' measure rather than global HRQoL. Possibly report item-total correlations to complement IRT."*

**Response:** We thank the reviewer for this important conceptual and methodological point. We agree that IRT results derived from a three-item scale should be interpreted with caution, and we have clarified this limitation more explicitly in the revised Discussion.

We also appreciate the suggestion to complement the IRT analyses with item-level indices from classical test theory. Corrected item–total correlations and inter-item correlations were already reported in the manuscript; however, we have now made their interpretive relevance more explicit. Specifically, the corrected item–total correlations were high for all three items (Symptoms: ρ = 0.788; Emotion: ρ = 0.840; Activity: ρ = 0.870; all p < .001), indicating that each item contributes substantially to the overall score. At the same time, the inter-item correlations were lower (ρ = 0.505–0.612), suggesting that the items are related but not redundant.

We also agree with the reviewer’s broader conceptual concern that, due to its ultra-brief format, the Skindex-Mini may not capture the full breadth of dermatology-specific HRQoL to the same extent as longer instruments. We have therefore revised the Discussion to clarify that the Skindex-Mini should be interpreted as an ultra-brief indicator of dermatology-related quality-of-life burden, with particular sensitivity to emotional and interpersonal impairment, rather than as a comprehensive assessment of all HRQoL domains. In line with this suggestion, we revised not only the relevant limitation paragraph, but also the Abstract and several statements in the Discussion, to ensure that the manuscript does not overstate the scope of the construct measured by the Skindex-Mini. The wording has been consistently tempered to reflect that the instrument functions as an ultra-brief indicator of dermatology-related burden, with particular sensitivity to emotional and interpersonal impairment, rather than as a fully comprehensive measure of all HRQoL domains.

**Location of changes:** *We have rewritten the Discussion section, Limitations subsection.*

*590-605 row*

*“Third, as identified through our IRT analysis, the Skindex-Mini demonstrates reduced measurement precision at higher levels of impairment. This suggests that while the instrument is excellent for screening and detecting mild-to-moderate HRQoL burden, it may be less sensitive to changes among patients with severe impairment—an important consideration when using the scale to monitor treatment response in severely affected populations. Although the three items cover symptom, emotional, and activity-related aspects of burden, the ultra-brief format necessarily limits content breadth. Accordingly, the Skindex-Mini should not be interpreted as a fully comprehensive representation of dermatology-specific HRQoL; rather, it may be best understood as a brief indicator of dermatology-related burden, with particular sensitivity to emotional and interpersonal impairment. Future research might explore whether this limitation could be addressed through the addition of one or two carefully selected items designed to capture the specific experiences of patients with profound HRQoL impairment, while maintaining the ultra-brief nature of the instrument. A modular approach could be considered, wherein the core three-item Skindex-Mini is administered universally, with optional follow-up items triggered only for patients who screen positive, allowing for more detailed assessment without burdening all patients.”*

**Comment 2.3:** *"Only SSCI-8 reaches that threshold (r = .43). Please would you consider discussing this? 'moderate-to-strong convergent validity' in the abstract seems overstated."*

**Response:**

We thank the reviewer for this helpful observation. We agree that the phrase “moderate-to-strong convergent validity” overstated the magnitude of the observed associations, given that most correlations were in the weak-to-moderate range and only the SSCI-8 reached r = .43.

We have therefore revised the Abstract [...] to use more cautious wording. Specifically, we now describe the findings as showing weak-to-moderate convergent validity, rather than moderate-to-strong convergent validity.

In addition, we now explicitly discuss the magnitude of these associations under the ***Accessing the discriminatory capacity of the Skindex across dermatological diagnoses*** sections and interpret them more cautiously.

**Location of change:** *Abstract and Discussion sections.*

- ***Abstract section***

***Despite****: „The instrument demonstrated weak-to-moderate convergent validity with measures of stigma and distress, and acceptable discriminant validity with measures of well-being.”*

***Revised change****: “The tool demonstrated* ***moderate*** *convergent validity with stigma and distress.”*

- ***Page 40. under Accessing the discriminatory capacity of the Skindex across dermatological diagnoses***

*„The analysis suggests that the Skindex scale shows weak to moderate ability to differentiate levels of quality-of-life impairment across various dermatological conditions.”*

**Location of change:** *Page 40. under the Accessing the discriminatory capacity of the Skindex across dermatological diagnoses sections*

*“**The analysis suggests that the Skindex scale shows weak to moderate ability to differentiate levels of quality-of-life impairment across various dermatological conditions.”*

**Comment 2.4:** *"The regression F(14,113) suggests only 128 cases were included. That is extremely small relative to 14 predictors. The power is limited."*

**Response:**

We thank the reviewer for this important methodological observation. We agree that the regression model was based on a limited effective sample size relative to the number of predictors, which restricts statistical power and may reduce the stability of coefficient estimates. We now acknowledge this explicitly in both the Results and the Limitations sections and clarify that these regression findings should be interpreted as exploratory rather than confirmatory.

**Location of change:**  *Results section – Hierarchical Regression Analysis*

*"**The demographic block did not significantly predict Skindex-Mini scores (p > .05). The addition of the psychometric block significantly improved model fit (F(14, 113) = 3.587, p < .001), with an adjusted R² of 0.222, indicating a small effect size. However, it should be noted that the final regression model included only 128 cases after listwise deletion, yielding a cases-to-predictors ratio of approximately 9:1. This falls below the commonly recommended threshold of 10:1 to 15:1 for stable regression estimates, and consequently the analysis may be underpowered to detect smaller effects. Findings from the regression should therefore be interpreted as exploratory and hypothesis-generating rather than confirmatory.”*

***2. Limitations section – Page 46; line: 606-612***

*"**Fourth, the hierarchical regression analysis was limited by a reduced effective sample size (N = 128 after listwise deletion) relative to the number of predictors (k = 14), resulting in a cases-to-predictors ratio of approximately 9:1. This raises concerns about statistical power and the stability of coefficient estimates. While multicollinearity was within acceptable limits (VIF range = 1.201–3.871) and model diagnostics were satisfactory, the risk of Type II errors cannot be excluded. Future studies with larger samples and lower rates of missing data are needed to confirm the independent predictors identified here."*

**Comment 2.5:** *"The instruments are barely described. For a reader unfamiliar with Hungarian versions or subscales, interpretation is impossible without supplements. PLOS ONE readers will not go to supplements to understand basic scale properties. They need: Scale range, Interpretation of higher/lower scores, Internal consistency in current sample, Sample item example (brief)."*

**Response:** We agree that the original manuscript did not provide enough information about the measures for readers unfamiliar with the Hungarian versions. We have therefore expanded the Instruments subsection in the main text. For each questionnaire included in the present analyses, we now report the number of items, response options, scoring procedure, interpretation of higher versus lower scores, internal consistency in the current sample, and a brief sample item where appropriate. This information is now available in the main manuscript rather than being left to the supplementary material..

**Location of change:** *Methods section, Instruments subsection – Page: 13.*

***“****To assess the construct validity of the Hungarian Skindex-Mini, participants completed a series of established, validated questionnaires covering health-related quality of life, emotion regulation, stigma, and psychological well-being. For each instrument described below, the internal consistency (Cronbach's α) was calculated for the current sample and is reported to provide context for the subsequent analyses.*

***Dermatology-Specific Quality of Life***

- ***Skindex-Mini QoL Health Questionnaire (Skindex-Mini)*** *[9] is a 3-item ultrashort measure designed to assess dermatology-specific health-related quality of life (HRQoL). Each item represents a core domain: symptom burden (e.g., "In the past week, how often were you bothered by your skin symptoms...?"), emotional impact (e.g., "...how often did your skin symptoms cause emotional difficulties...?"), and functional limitations (e.g., "...how often did your skin symptoms make it difficult for you to engage in activities...?"). Respondents rate the frequency of each experience over the past week on a 5-point Likert scale (1 = Never to 5 = All the time). Item scores are summed to produce a total raw score (range 3--15), which is then transformed to a 0--100 linear scale, with higher scores indicating greater impairment (worse QoL). The internal consistency in the current sample was good (Cronbach's α = 0.78).*
- ***Dermatology Life Quality Index-Revised (DLQI-R)*** *[7, 8] is a 10-item questionnaire that assesses the impact of skin diseases on QoL over the past week. It covers domains such as symptoms and feelings, daily activities, leisure, work or school, personal relationships, and treatment. Items are rated on a 4-point Likert scale (0 = Not at all/Not relevant to 3 = Very much). A sample item is: "Over the last week, how itchy, sore, painful or stinging has your skin been?" The total score ranges from 0 to 30, with higher scores indicating greater QoL impairment. The DLQI-R was used to assess known-groups validity and as an anchor for determining clinically significant cut-points on the Skindex-Mini. In this sample, the DLQI-R demonstrated excellent internal consistency (α = 0.90).*

***Emotion Regulation and Distress***

- ***Difficulties in Emotion Regulation Scale-16 (DERS-16)*** *[14, 15] is a 16-item self-report measure that assesses clinically relevant difficulties in emotion regulation. It comprises five subscales: Non-acceptance of emotional responses, Difficulty engaging in goal-directed behavior, Impulse control difficulties, Limited access to emotion regulation strategies, and Lack of emotional clarity. Items (e.g., "I have difficulty making sense out of my feelings") are rated on a 5-point Likert scale (1 = Almost never to 5 = Almost always). Total and subscale scores are calculated as the mean of the respective items, with higher scores indicating greater difficulties in emotion regulation. Internal consistency in our sample was excellent for the total score (α = 0.95) and good-to-excellent for the subscales (α range = 0.85--0.94).*
- ***9-item Beck Depression Inventory-Fast Screen (BDI-FS)*** *[19, 20] is a 9-item screening tool for depressive symptoms in medical patients, excluding somatic items that might be confounded with physical illness. Items (e.g., "Sadness") are rated on a 4-point scale (0 to 3), with total scores ranging from 0 to 27. Higher scores indicate more severe depressive symptoms. The internal consistency in this sample was acceptable (α = 0.74).*
- ***Distress Thermometer (DT)*** *[18, 26] is a single-item, 11-point visual analog scale (0 = No distress to 10 = Extreme distress) that asks patients to rate their average level of psychological distress over the past week, including the current day. Higher scores indicate greater distress. It is widely used in psycho-oncology and psychodermatology as a rapid screening tool. In this study, it was used to assess convergent validity (α is not applicable for a single-item measure).*

***Stigma and Social Functioning***

- ***Stigmatization Scale for Chronic Illnesses--8 Questionnaire (SSCI-8)*** *[16] is an 8-item measure that assesses perceived or internalized stigma related to having a chronic illness. Items (e.g., "Because of my illness, I felt left out of things") are rated on a 5-point Likert scale (1 = Never to 5 = Always). A total score is calculated as the mean of all items, with higher scores indicating a greater degree of perceived stigmatization. The scale showed good internal consistency in the current sample (α = 0.89).*
- ***Body Absorption Scale (BAS)*** *[24] is a 10-item instrument that measures an individual's tendency to focus deeply on internal bodily sensations. Items (e.g., "If there is something wrong with my body, I can feel it immediately") are rated on a 5-point Likert scale (1 = Not true to 5 = Very true). A total score is calculated as the mean of all items, with higher scores indicating greater body absorption. It was included to assess discriminant validity, as it is conceptually distinct from HRQoL. In this sample, the BAS demonstrated acceptable internal consistency (α = 0.73).*

***Psychological Well-Being and Anger Expression***

- ***WHO-5 Well-Being Index (WBI-5)*** *[21, 22] is a 5-item scale that measures positive psychological well-being. Items (e.g., "I have felt cheerful and in good spirits") refer to the previous two weeks and are rated on a 6-point Likert scale (0 = At no time to 5 = All of the time). The raw total score (range 0--25) is multiplied by 4 to yield a percentage score from 0 to 100, with higher scores indicating better well-being. It was used to assess divergent validity. The internal consistency was good in this sample (α = 0.83).*
- ***Anger Expression Scale (AX Scale)*** *[23] is a widely used measure of anger expression. For this study, we used two subscales: Anger-In (suppression of angry feelings) and Anger-Out (expression of anger towards others or objects). Items are rated on a 4-point Likert scale (1 = Almost never to 4 = Almost always). Subscale scores are calculated as the mean of their respective items, with higher scores indicating a greater tendency to suppress (Anger-In) or express (Anger-Out) anger. These subscales were used to assess convergent (Anger-In) and discriminant (Anger-Out) validity. In our sample, both subscales showed acceptable internal consistency (Anger-In: α = 0.76; Anger-Out: α = 0.70).*

***General Health Status***

Visual Analog Scale (EQ-VAS) of the EuroQol-5D Scale (EQ-5D) [8, 25] is a standard vertical 20 cm visual analogue scale for recording an individual's self-rated health on a scale from 0 ("The worst health you can imagine") to 100 ("The best health you can imagine"). It provides a simple, global measure of current health status. Higher scores indicate better perceived health.”

**Comment 2.6:** *"Gender percentages are presented awkwardly. They should be column percentages within sample, not mixed formats. Also, 81% women — that requires Discussion. Sampling bias is barely addressed."*

**Response:** We thank the reviewer for this important methodological observation. We have addressed both aspects of this comment.

First, regarding the presentation of gender percentages, we have revised the text. The gender distribution is now reported clearly, with percentages calculated separately for Sample 1, Sample 2.

## **Location of change:** Description of the sample

“*Valid gender data were available for 560 participants. In the combined sample, 117 participants were male (20.9%), 438 were female (78.2%), and 5 (0.9%) indicated another gender category. Because responses were not mandatory, the number of valid observations varied across variables.”*

Second, we agree with the reviewer that the substantial overrepresentation of female participants (81% of the combined sample) requires explicit Discussion as a potential source of sampling bias. We have now expanded the Limitations section to address this point directly. Specifically, we acknowledge that women typically report higher levels of health-related quality of life impairment and may differ in emotion regulation patterns and help-seeking behaviors compared to men, which may influence the generalizability of our findings. We note that while the gender distribution in our sample reflects the typical overrepresentation of women in dermatology outpatient populations and psychodermatology research, future studies should aim for more balanced recruitment to confirm the measurement invariance of the Skindex-Mini across genders.

**Location of change:** *Discussion section, Limitations subsection – Page 44.; line: 570-579.*

*“****Limitations and Future Directions***

*Second, the sample was characterized by a high proportion of female respondents (81%), which may affect generalizability. Research consistently indicates that women tend to report higher HRQoL impairment and may differ in emotion regulation patterns and illness perception compared to men; thus, the psychometric performance observed here should be confirmed in more gender-balanced cohorts. Future validation studies should also prioritize recruitment of more diverse clinical populations, including community-based samples, patients managed exclusively in primary care settings (who may have milder disease), and those requiring hospitalization for severe dermatological conditions. Multi-center studies incorporating these diverse care settings would substantially strengthen the generalizability of our findings.”*
