# Supplementary material for: Reliability and construct validity of the Hungarian version of Skindex-Mini
Source: PLoS One. 2026 Jun 23;21(6):e0350749. doi: 10.1371/journal.pone.0350749 (PMC13289942; doi:10.1371/journal.pone.0350749)
Supplement: Peer review history

# Peer review: Decision Letter 0

**DOI:** 10.1371/journal.pone.0350749.r001

**Authors:** Pavel Chernyshov

2 Mar 2026

PONE-D-26-03636

Reliability and Construct validity of the Hungarian version of Skindex‐Mini

PLOS One

Dear Dr. Német,

Thank you for submitting your manuscript to PLOS ONE. After careful consideration, we feel that it has merit but does not fully meet PLOS ONE’s publication criteria as it currently stands. Therefore, we invite you to submit a revised version of the manuscript that addresses the points raised during the review process.

Please submit your revised manuscript by Apr 16 2026 11:59PM. If you will need more time than this to complete your revisions, please reply to this message or contact the journal office at plosone@plos.org. When you’re ready to submit your revision, log on to https://www.editorialmanager.com/pone/ and select the 'Submissions Needing Revision' folder to locate your manuscript file.

Please include the following items when submitting your revised manuscript:

- A letter that responds to each point raised by the academic editor and reviewer(s). You should upload this letter as a separate file labeled 'Response to Reviewers'.
- A marked-up copy of your manuscript that highlights changes made to the original version. You should upload this as a separate file labeled 'Revised Manuscript with Track Changes'.
- An unmarked version of your revised paper without tracked changes. You should upload this as a separate file labeled 'Manuscript'.

If you would like to make changes to your financial disclosure, please include your updated statement in your cover letter. Guidelines for resubmitting your figure files are available below the reviewer comments at the end of this letter.

If applicable, we recommend that you deposit your laboratory protocols in protocols.io to enhance the reproducibility of your results. Protocols.io assigns your protocol its own identifier (DOI) so that it can be cited independently in the future. For instructions see: https://journals.plos.org/plosone/s/submission-guidelines#loc-laboratory-protocols. Additionally, PLOS ONE offers an option for publishing peer-reviewed Lab Protocol articles, which describe protocols hosted on protocols.io. Read more information on sharing protocols at https://plos.org/protocols?utm_medium=editorial-email&utm_source=authorletters&utm_campaign=protocols.

We look forward to receiving your revised manuscript.

Kind regards,

Pavel V. Chernyshov, M.D., Ph.D.

Academic Editor

PLOS One

Journal Requirements:

When submitting your revision, we need you to address these additional requirements.

1. Please ensure that your manuscript meets PLOS ONE's style requirements, including those for file naming. The PLOS ONE style templates can be found at

https://journals.plos.org/plosone/s/file?id=wjVg/PLOSOne_formatting_sample_main_body.pdf and

https://journals.plos.org/plosone/s/file?id=ba62/PLOSOne_formatting_sample_title_authors_affiliations.pdf

2.  Please include your full ethics statement in the ‘Methods’ section of your manuscript file. In your statement, please include the full name of the IRB or ethics committee who approved or waived your study, as well as whether or not you obtained informed written or verbal consent. If consent was waived for your study, please include this information in your statement as well.

3. If the reviewer comments include a recommendation to cite specific previously published works, please review and evaluate these publications to determine whether they are relevant and should be cited. There is no requirement to cite these works unless the editor has indicated otherwise.

[Note: HTML markup is below. Please do not edit.]

Reviewers' comments:

Reviewer's Responses to Questions

**Comments to the Author**

1. Is the manuscript technically sound, and do the data support the conclusions?

The manuscript must describe a technically sound piece of scientific research with data that supports the conclusions. Experiments must have been conducted rigorously, with appropriate controls, replication, and sample sizes. The conclusions must be drawn appropriately based on the data presented.

Reviewer #1: Yes

Reviewer #2: Yes

**********

2. Has the statistical analysis been performed appropriately and rigorously?

Reviewer #1: Yes

Reviewer #2: Yes

**********

3. Have the authors made all data underlying the findings in their manuscript fully available?

The PLOS Data policy requires authors to make all data underlying the findings described in their manuscript fully available without restriction, with rare exception (please refer to the Data Availability Statement in the manuscript PDF file). The data should be provided as part of the manuscript or its supporting information, or deposited to a public repository. For example, in addition to summary statistics, the data points behind means, medians and variance measures should be available. If there are restrictions on publicly sharing data—e.g. participant privacy or use of data from a third party—those must be specified.

Reviewer #1: Yes

Reviewer #2: Yes

**********

4. Is the manuscript presented in an intelligible fashion and written in standard English?

PLOS ONE does not copyedit accepted manuscripts, so the language in submitted articles must be clear, correct, and unambiguous. Any typographical or grammatical errors should be corrected at revision, so please note any specific errors here.

Reviewer #1: Yes

Reviewer #2: Yes

**********

5. Review Comments to the Author

Please use the space provided to explain your answers to the questions above. You may also include additional comments for the author, including concerns about dual publication, research ethics, or publication ethics. (Please upload your review as an attachment if it exceeds 20,000 characters)

Reviewer #1: This manuscript presents a solid psychometric validation of the Hungarian Skindex-Mini. The study is original, clinically relevant, and methodologically sound.

Major points:

The Discussion section could benefit from a more in-depth interpretation of the findings. Specifically, the authors could elaborate on why certain items (e.g., SM3) demonstrate high discriminative ability and discuss the practical implications for ultra-brief screening in clinical settings.

Additionally, although the Introduction provides a detailed comparison with previous validation studies across languages and conditions, the Discussion does not fully contextualize the present findings within this broader literature, such as similarities or differences in performance across dermatological populations.

Limitations should be mentioned, such as the high proportion of female respondents, potential recruitment bias, and reduced precision at higher levels of impairment.

The authors could also expand on clinical implications, such as how the cut-off scores could be used in practice, how the Skindex-Mini could inform treatment decisions or monitor response to interventions, and the possible use in teledermatology or primary care.

They could suggest directions for future validation, e.g., longitudinal studies to assess responsiveness to change, studies in more diverse clinical populations, or adding items to improve measurement at severe impairment levels.

Minor points:

In the Methods it would be useful to include a brief description of the Skindex-Mini and its scoring.

In the note of Table 4 it is written “All correlations are Spearman’s rho”. However, there are no correlations in the table.

Reviewer #2: This is a solid psychometric validation paper of the Hungarian version of Skindex Mini. The paper is based on a large sample, multiple constructs, CFA, IRT, ROC and hierarchical regression increase its value.

The statistics are sound, but I have some minor comments:

- Using KMO and Bartlett in a CFA framework is conceptually inconsistent.

- IRT with 3 items is unstable and clinically limited. This is acknowledged as a limitation. I sugegst you consider addressing whether the scale is functionally an “emotional impairment” measure rather than global HRQoL. Possibly report item-total correlations to complement IRT

- Only SSCI-8 reaches that threshold (r = .43). Please would you consider discussing this? 'moderate-to-strong convergent validity' in the abstract seems overstated.

- The regression F(14,113) suggests only 128 cases were included. That is extremely small relative to 14 predictors. The power is limited.

The instruments are barely described. For a reader unfamiliar with Hungarian versions or subscales, interpretation is impossible without supplements. PLOS ONE readers will not go to supplements to understand basic scale properties. They need: Scale range, Interpretation of higher/lower scores, Internal consistency in current sample, Sample item example (brief)

Gender percentages are presented awkwardly. They should be column percentages within sample, not mixed formats. Also, 81% women — that requires discussion. It may bias QoL severity and emotion regulation findings. Sampling bias is barely addressed.

Recruitement lasted 4 years.Covid-related psychological burden is not discussed.

alpha = 0.78–0.80, that’s good. But alpha is inflated by item covariance. With only 3 items, internal consistency is constrained. Reporting alpha as strong psychometric support should be interpreted cautiously. The authors repeatedly describe the properties of Skindex-Mini as “robust.” This is overused. This is a brief screening tool with acceptable psychometrics — not a robust multidimensional instrument.

Minor:

Table titles should be improved to reflect what is being described. Titles are too generic and can not be uderstood as standalone.

In Conclusion, I suggest you discuss Clinical relevance of Skindex-Mini, Interpretability for dermatologists and whether the instrument adds something meaningful beyond DLQI.

**********

6. PLOS authors have the option to publish the peer review history of their article (what does this mean?). If published, this will include your full peer review and any attached files.

If you choose “no”, your identity will remain anonymous but your review may still be made public.

**Do you want your identity to be public for this peer review?**  For information about this choice, including consent withdrawal, please see our Privacy Policy.

Reviewer #1: No

Reviewer #2: **Yes:** Flora Balieva

**********

[NOTE: If reviewer comments were submitted as an attachment file, they will be attached to this email and accessible via the submission site. Please log into your account, locate the manuscript record, and check for the action link "View Attachments". If this link does not appear, there are no attachment files.]

To ensure your figures meet our technical requirements, please review our figure guidelines: https://journals.plos.org/plosone/s/figures

You may also use PLOS’s free figure tool, NAAS, to help you prepare publication quality figures: https://journals.plos.org/plosone/s/figures#loc-tools-for-figure-preparation.

NAAS will assess whether your figures meet our technical requirements by comparing each figure against our figure specifications.

# Author comment: Author response to Decision Letter 1

**DOI:** 10.1371/journal.pone.0350749.r002

1 May 2026

Response to Reviewers

Manuscript ID: PONE-D-26-03636

Title: Reliability and Construct Validity of the Hungarian Version of Skindex-Mini

Journal: PLOS One

Corresponding Author: Borbála Német

Dear Dr. Chernyshov and Reviewers,

We sincerely thank you for the opportunity to revise our manuscript entitled "Reliability and Construct Validity of the Hungarian Version of Skindex-Mini." We are grateful to the Academic Editor and the reviewers for their thoughtful and constructive comments, which have helped us substantially improve the quality of our work.

We have carefully addressed all points raised in the review process. Below, we provide a point-by-point response to each comment, describing the changes made to the manuscript. All modifications have been marked with track changes in the revised manuscript file.

Response to Reviewer #1

We thank Reviewer #1 for the positive assessment of our work and the constructive suggestions for improvement.

Comment 1.1: "The Discussion section could benefit from a more in-depth interpretation of the findings. Specifically, the authors could elaborate on why certain items (e.g., SM3) demonstrate high discriminative ability and discuss the practical implications for ultra-brief screening in clinical settings."

Response: We agree with this valuable suggestion. We have expanded the Discussion section to include a more detailed interpretation of item-level findings. Specifically, we have added a paragraph elaborating on why item SM3 ("Your skin condition made it difficult to show affection") demonstrated particularly high discriminative ability, noting that this item captures the interpersonal and emotional consequences of skin diseases that may be especially salient in conditions affecting visible areas. We also discuss how this finding aligns with previous research highlighting the social burden of dermatological conditions. Additionally, we have elaborated on the practical implications of the Skindex-Mini as an ultra-brief screening tool, emphasizing its utility in time-constrained clinical settings, its potential for routine use to identify patients in need of psychosocial support, and its ease of administration in both outpatient and teledermatology contexts.

Location of change: Discussion section.

Comment 1.2: "Although the Introduction provides a detailed comparison with previous validation studies across languages and conditions, the Discussion does not fully contextualize the present findings within this broader literature, such as similarities or differences in performance across dermatological populations."

Response: We thank the reviewer for highlighting this important gap. We have substantially revised the Discussion to better contextualize our findings within the broader literature on Skindex-Mini validations. We now explicitly compare our psychometric findings (internal consistency, factor structure, convergent validity) with those reported in previous validation studies from other linguistic and cultural contexts (e.g., German, Italian, Spanish, Chinese versions). We discuss how the Hungarian version performs similarly to other adaptations in terms of reliability and validity, while noting that slight variations may be attributable to sample characteristics (e.g., diagnostic composition) or cultural factors in health-related quality of life reporting.

Location of change: We have rewritten the Discussion section.

Comment 1.3: "Limitations should be mentioned, such as the high proportion of female respondents, potential recruitment bias, and reduced precision at higher levels of impairment."

Response: We appreciate this important observation. We have significantly expanded the Limitations section to address these points explicitly. We now acknowledge the overrepresentation of female participants (81%) as a limitation that may affect generalizability, as women typically report higher HRQoL impairment and may differ in emotion regulation patterns. We also discuss potential recruitment bias stemming from the single-center, outpatient clinic setting, which may not represent the full spectrum of dermatological patients (e.g., those with milder conditions managed in primary care or severe cases requiring hospitalization). Additionally, we note the reduced precision of the Skindex-Mini at higher levels of impairment as identified through our IRT analysis, suggesting that the instrument may be less sensitive to changes among patients with severe HRQoL burden—an important consideration for monitoring treatment response in severely affected populations.

Location of change: We have rewritten the Discussion section, Limitations subsection.

“Limitations and Future Directions

Second, the sample was characterized by a high proportion of female respondents (81%), which may affect generalizability. Research consistently indicates that women tend to report higher HRQoL impairment and may differ in emotion regulation patterns and illness perception compared to men; thus, the psychometric performance observed here should be confirmed in more gender-balanced cohorts. Future validation studies should also prioritize recruitment of more diverse clinical populations, including community-based samples, patients managed exclusively in primary care settings (who may have milder disease), and those requiring hospitalization for severe dermatological conditions. Multi-center studies incorporating these diverse care settings would substantially strengthen the generalizability of our findings.”

Comment 1.4: "The authors could also expand on clinical implications, such as how the cut-off scores could be used in practice, how the Skindex-Mini could inform treatment decisions or monitor response to interventions, and the possible use in teledermatology or primary care."

Response: We thank the reviewer for this practical focus. We have added a new subsection on "Clinical Implications" within the Discussion. In this section, we elaborate on how the established cut-off scores (using the 75th percentile as a threshold for clinically significant impairment) can be readily implemented in routine practice to identify patients who may benefit from additional psychosocial support or referral. We discuss how the Skindex-Mini can complement clinical assessment by providing a patient-reported outcome measure that captures the subjective burden of skin disease, potentially informing treatment decisions (e.g., escalation of therapy when HRQoL impairment is severe) and monitoring response to interventions over time. We also highlight the particular utility of this ultra-brief instrument in teledermatology consultations and busy primary care settings where time is limited, enabling systematic screening for HRQoL impairment that might otherwise be overlooked.

Location of change: Discussion section, Clinical Implications subsection

“Clinical Implications

The robust psychometric properties of the Hungarian Skindex-Mini, combined with its three-item format, have significant practical implications for routine clinical care. Its ultra-brief nature makes it exceptionally well-suited for time-constrained settings where longer questionnaires are impractical, including busy outpatient clinics, primary care practices, and teledermatology consultations. It can be administered, scored, and interpreted in under a minute, allowing for routine quality-of-life screening during every patient visit without disrupting clinic workflow.

The establishment of cut-off scores enhances the clinical utility of the instrument. Based on our analysis, a Skindex-Mini score of ≥13 (corresponding to the 75th percentile) represents a clinically meaningful threshold for identifying patients experiencing significant psychosocial burden. In practice, this cut-off can function as a "red flag," prompting clinicians to initiate further Discussion about the emotional and social impact of the skin condition, explore the need for psychosocial support, or consider referral to a psychodermatology specialist. Conversely, the high negative predictive value of the instrument means that scores below this threshold can reliably reassure clinicians that significant HRQoL impairment is unlikely, allowing consultation time to be focused on other aspects of care.

Beyond screening, the Skindex-Mini can inform treatment decisions and monitor response to interventions over time. By providing a standardized patient-reported outcome measure that captures the subjective burden of skin disease, the instrument complements objective clinical assessments (e.g., physician-rated severity scores) that may not fully reflect the patient's lived experience. For instance, a patient with objectively mild psoriasis but a Skindex-Mini score above the clinical threshold may warrant more aggressive treatment or adjunctive psychological support than disease severity alone would suggest. Conversely, a patient whose clinical severity remains stable but whose Skindex-Mini score shows sustained improvement following an intervention provides valuable evidence of treatment effectiveness from the patient's perspective. When administered longitudinally—such as at each follow-up visit—the Skindex-Mini can track changes in HRQoL over time, offering a sensitive measure of response to pharmacological, surgical, or psychological interventions.

The instrument's brevity and simplicity also enhance its utility in emerging healthcare delivery models. In teledermatology, where non-verbal cues may be less accessible to clinicians, the Skindex-Mini can be easily integrated into electronic intake forms or sent to patients prior to virtual consultations, ensuring that the patient's perspective on their HRQoL is systematically captured. Similarly, in primary care settings—where many dermatological conditions are initially managed but dermatology expertise may be limited—the Skindex-Mini provides a practical tool for identifying patients whose quality-of-life impairment warrants specialist referral. By making standardized QoL assessment feasible across diverse healthcare contexts, the Skindex-Mini can bridge the gap between recognizing the psychosocial burden of skin disease and actively monitoring and addressing it in clinical care.”

Comment 1.5: "They could suggest directions for future validation, e.g., longitudinal studies to assess responsiveness to change, studies in more diverse clinical populations, or adding items to improve measurement at severe impairment levels."

Response: We agree and have expanded the Future Directions section accordingly. The revised Discussion now highlights the need for longitudinal studies examining responsiveness to change, validation in more diverse clinical populations, and possible strategies for improving measurement precision at the severe end of the burden spectrum while preserving the instrument’s brevity.

Location of change: Discussion section, Limitations and Future Directions subsection.

“Limitations and Future Directions

The findings of this study should be interpreted in light of several limitations, which also point toward important directions for future research. First, the cross-sectional design precludes analysis of the Skindex-Mini's responsiveness to change—a key attribute for a tool intended to monitor treatment efficacy over time. Longitudinal studies are urgently needed to establish the instrument's sensitivity in detecting clinically meaningful improvements following pharmacological, surgical, or psychological interventions. Such studies would also enable the determination of minimal clinically important differences (MCID), further enhancing the utility of the Skindex-Mini as an outcome measure in both clinical practice and interventional research.

Third, as identified through our IRT analysis, the Skindex-Mini demonstrates reduced measurement precision at higher levels of impairment. This suggests that while the instrument is excellent for screening and detecting mild-to-moderate HRQoL burden, it may be less sensitive to changes among patients with severe impairment—an important consideration when using the scale to monitor treatment response in severely affected populations. Future research might explore whether this limitation could be addressed through the addition of one or two carefully selected items designed to capture the specific experiences of patients with profound HRQoL impairment, while maintaining the ultra-brief nature of the instrument. A modular approach could be considered, wherein the core three-item Skindex-Mini is administered universally, with optional follow-up items triggered only for patients who screen positive, allowing for more detailed assessment without burdening all patients.

Finally, while the high negative predictive value of the Skindex-Mini makes it suitable for rapid clinical screening, its performance in multinational research contexts should continue to be evaluated to ensure cross-cultural comparability. Comparative studies across different linguistic and cultural versions of the Skindex-Mini would help establish whether the instrument functions equivalently across populations, enabling its use in international trials and cross-cultural epidemiological research.

In conclusion, the Hungarian Skindex-Mini is a psychometrically robust, ultra-brief instrument that provides a valid and reliable indicator of dermatology-related burden, although its brevity necessarily limits the breadth of HRQoL content captured. Its strong performance, combined with its exceptional feasibility, positions it as a valuable tool for both clinical practice and research, capable of bringing the patient's voice into routine dermatologic care.”

Comment 1.6: "In the Methods it would be useful to include a brief description of the Skindex-Mini and its scoring."

Response: We agree that this information is essential for readers unfamiliar with the instrument. We have added a brief description of the Skindex-Mini to the Methods section, Instruments subsection, including the number of items (3), the response scale (5-point Likert scale from "never" to "all the time"), the scoring method (summed raw scores converted to a 0-100 linear scale), and interpretation (higher scores indicate greater HRQoL impairment). We also note the domains covered (symptoms, emotions, social functioning) as represented by the three items.

Location of change: Methods section, Instruments subsection

“Instruments

To assess the construct validity of the Hungarian Skindex-Mini, participants completed a series of established, validated questionnaires covering health-related quality of life, emotion regulation, stigma, and psychological well-being. For each instrument described below, the internal consistency (Cronbach's α) was calculated for the current sample and is reported to provide context for the subsequent analyses.

Dermatology-Specific Quality of Life

• Skindex-Mini QoL Health Questionnaire (Skindex-Mini) [9] is a 3-item ultrashort measure designed to assess dermatology-specific health-related quality of life (HRQoL). Each item represents a core domain: symptom burden (e.g., "In the past week, how often were you bothered by your skin symptoms...?"), emotional impact (e.g., "...how often did your skin symptoms cause emotional difficulties...?"), and functional limitations (e.g., "...how often did your skin symptoms make it difficult for you to engage in activities...?"). Respondents rate the frequency of each experience over the past week on a 5-point Likert scale (1 = Never to 5 = All the time). Item scores are summed to produce a total raw score (range 3--15), which is then transformed to a 0--100 linear scale, with higher scores indicating greater impairment (worse QoL). The internal consistency in the current sample was good (Cronbach's α = 0.78).”

Comment 1.7: "In the note of Table 4 it is written 'All correlations are Spearman's rho'. However, there are no correlations in the table."

Response: We thank the reviewer for identifying this error. Table 4 presents regression results, not correlations. The note was incorrectly retained from an earlier version of the manuscript. We have removed this erroneous note from Table 4 and ensured that the table title and notes accurately reflect the content (hierarchical regression analysis).

Location of change:

Attachment Submitted filename: Response to Reviewers.docx

# Peer review: Decision Letter 1

**DOI:** 10.1371/journal.pone.0350749.r003

**Authors:** Pavel Chernyshov

19 May 2026

Reliability and Construct validity of the Hungarian version of Skindex‐Mini

PONE-D-26-03636R1

Dear Dr. Német,

We’re pleased to inform you that your manuscript has been judged scientifically suitable for publication and will be formally accepted for publication once it meets all outstanding technical requirements.

Within one week, you’ll receive an e-mail detailing the required amendments. When these have been addressed, you’ll receive a formal acceptance letter and your manuscript will be scheduled for publication.

An invoice will be generated when your article is formally accepted. Please note, if your institution has a publishing partnership with PLOS and your article meets the relevant criteria, all or part of your publication costs will be covered. Please make sure your user information is up-to-date by logging into Editorial Manager at Editorial Manager® and clicking the ‘Update My Information' link at the top of the page. For questions related to billing, please contact billing support.

If your institution or institutions have a press office, please notify them about your upcoming paper to help maximize its impact. If they’ll be preparing press materials, please inform our press team as soon as possible -- no later than 48 hours after receiving the formal acceptance. Your manuscript will remain under strict press embargo until 2 pm Eastern Time on the date of publication. For more information, please contact onepress@plos.org.

Kind regards,

Pavel V. Chernyshov, M.D., Ph.D.

Academic Editor

PLOS One

Additional Editor Comments (optional):

Reviewers' comments:

Reviewer's Responses to Questions

**Comments to the Author**

1. If the authors have adequately addressed your comments raised in a previous round of review and you feel that this manuscript is now acceptable for publication, you may indicate that here to bypass the “Comments to the Author” section, enter your conflict of interest statement in the “Confidential to Editor” section, and submit your "Accept" recommendation.

Reviewer #1: All comments have been addressed

Reviewer #2: All comments have been addressed

**********

2. Is the manuscript technically sound, and do the data support the conclusions?

The manuscript must describe a technically sound piece of scientific research with data that supports the conclusions. Experiments must have been conducted rigorously, with appropriate controls, replication, and sample sizes. The conclusions must be drawn appropriately based on the data presented.

Reviewer #1: Yes

Reviewer #2: Yes

**********

3. Has the statistical analysis been performed appropriately and rigorously?

Reviewer #1: Yes

Reviewer #2: Yes

**********

4. Have the authors made all data underlying the findings in their manuscript fully available?

The PLOS Data policy requires authors to make all data underlying the findings described in their manuscript fully available without restriction, with rare exception (please refer to the Data Availability Statement in the manuscript PDF file). The data should be provided as part of the manuscript or its supporting information, or deposited to a public repository. For example, in addition to summary statistics, the data points behind means, medians and variance measures should be available. If there are restrictions on publicly sharing data—e.g. participant privacy or use of data from a third party—those must be specified.

Reviewer #1: Yes

Reviewer #2: Yes

**********

5. Is the manuscript presented in an intelligible fashion and written in standard English?

PLOS ONE does not copyedit accepted manuscripts, so the language in submitted articles must be clear, correct, and unambiguous. Any typographical or grammatical errors should be corrected at revision, so please note any specific errors here.

Reviewer #1: Yes

Reviewer #2: Yes

**********

6. Review Comments to the Author

Please use the space provided to explain your answers to the questions above. You may also include additional comments for the author, including concerns about dual publication, research ethics, or publication ethics. (Please upload your review as an attachment if it exceeds 20,000 characters)

Reviewer #1: The authors have addressed all comments in a thorough manner and implemented the necessary revisions.

Reviewer #2: all comments and suggestions rom all reviewers have been adequately addressed and the paper is improved

**********

7. PLOS authors have the option to publish the peer review history of their article (what does this mean?). If published, this will include your full peer review and any attached files.

If you choose “no”, your identity will remain anonymous but your review may still be made public.

**Do you want your identity to be public for this peer review?**  For information about this choice, including consent withdrawal, please see our Privacy Policy.

Reviewer #1: No

Reviewer #2: **Yes:** Flora Balieva

**********

# Editor report: Acceptance letter

**DOI:** 10.1371/journal.pone.0350749.r004

**Authors:** Pavel Chernyshov

PONE-D-26-03636R1

PLOS One

Dear Dr. Német,

I'm pleased to inform you that your manuscript has been deemed suitable for publication in PLOS One. Congratulations! Your manuscript is now being handed over to our production team.

At this stage, our production department will prepare your paper for publication. This includes ensuring the following:

* All references, tables, and figures are properly cited

* All relevant supporting information is included in the manuscript submission,

* There are no issues that prevent the paper from being properly typeset

You will receive further instructions from the production team, including instructions on how to review your proof when it is ready. Please keep in mind that we are working through a large volume of accepted articles, so please give us a few days to review your paper and let you know the next and final steps.

Lastly, if your institution or institutions have a press office, please let them know about your upcoming paper now to help maximize its impact. If they'll be preparing press materials, please inform our press team within the next 48 hours. Your manuscript will remain under strict press embargo until 2 pm Eastern Time on the date of publication. For more information, please contact onepress@plos.org.

You will receive an invoice from PLOS for your publication fee after your manuscript has reached the completed accept phase. If you receive an email requesting payment before acceptance or for any other service, this may be a phishing scheme. Learn how to identify phishing emails and protect your accounts at https://explore.plos.org/phishing.

If we can help with anything else, please email us at customercare@plos.org.

Thank you for submitting your work to PLOS ONE and supporting open access.

Kind regards,

PLOS ONE Editorial Office Staff

on behalf of

Dr. Pavel V. Chernyshov

Academic Editor

PLOS One
